# Plastic cannulas mitigate arteriovenous fistula stenosis by suppressing the CFB-mediated inflammatory cascade

**DOI:** 10.3389/fimmu.2025.1715417

**Published:** 2025-12-08

**Authors:** Dongjuan Zhang, Deyang Kong, MingMing Zhao, Zhanci Ou, Lu Ma, Ka Qi, Yang Yang

**Affiliations:** 1 th Haemodialysis Centre, The 981^st^ Hospital of Joint Logistic Support Force, Chengde, China; 2 Department of Nephrology, Shenzhen Bao’an District Song Gang People’s Hospital, Shenzhen, China; 3 Hemodialysis Center, Sinopharm Harbin General Hospital, Harbin, China; 4 Department of Pathology, Shenzhen Bao’an District Song Gang People’s Hospital, Shenzhen, China; 5 Department of Nephrology, Beidaihe Rehabilitation and Recuperation Center of Chinese People’s Liberation Army, Qinhuangdao, China; 6 Yichen Hemodialysis Center, Qinhuangdao, China

**Keywords:** arteriovenous fistula, complement, early cannulation, failure, cannulation device

## Abstract

**Background:**

The choice of an appropriate cannulation technique should be important to increase the possibility of better outcomes in terms of arteriovenous fistulas (AVF) survival and comfort of the patient undergoing hemodialysis.

**Methods:**

It is a retrospective study and microarray analysis was conducted to identify differentially expressed genes (DEGs) between failing and control access samples. Sixty-four patients who underwent early cannulation (3–4 weeks after AVF creation) were enrolled and divided into two groups: a plastic cannula group (n = 33) and a metal needle group (n = 31). Comparisons were made between the groups regarding complement components, blood flow, access intimal hyperplasia, and inflammatory cell infiltration.

**Results:**

(1) AVF failure occurred in 13 patients (20.3%) over a mean follow-up of 241 ± 105 days. (2) Complement B factor (CFB) levels showed significant changes within the first two weeks post-cannulation. (3) Fluctuations in CFB strongly correlated with changes in AVF blood flow during follow-up. (4) CFB variation independently predicted AVF failure, with a hazard ratio of 4.54 (95% CI, 1.21–16.99). (5) The plastic cannula group exhibited significantly lower CFB expression in both blood and outflow access, along with marked improvements in intimal hyperplasia and inflammatory cell infiltration. (6) Compared with the metal needle group, serum from the plastic cannula more significantly induced endothelial cell proliferation and nitric oxide production, with CFB playing a critical role.

**Conclusions:**

The alternative complement pathway is significantly activated during initial AVF cannulation, with excessive CFB production contributing substantially to AVF failure. The use of plastic cannulas may improve long-term AVF patency by mitigating endothelial dysfunction and inhibiting inflammatory cell infiltration through suppression of CFB generation.

The autologous arteriovenous fistula (AVF) is established as the gold standard vascular access for hemodialysis (HD), as endorsed by current clinical guidelines (1, 2). However, the maturation and long-term maintenance of AVFs remain clinically challenging. Underlying this challenge are complex molecular pathologies. Transcriptomic analyses of human AVF tissues have revealed profound dysregulation, including altered expression of long non-coding RNAs (3) and microRNAs functionally linked to critical pathways such as MAPK signaling (4). Furthermore, innovative single-cell studies employing endovascular biopsy have directly demonstrated gene-specific dysregulation and phenotypic heterogeneity of endothelial cells in stenotic AVFs (5). Extending these findings to specific clinical contexts, a recent systems biology study identified shared pathogenic mechanisms and key biomarkers, such as HPGD, between diabetic kidney disease (DKD) and AVF stenosis, highlighting the role of metabolic-immune dysregulation in this process (6). These findings collectively paint a picture of AVF failure as a state of sustained transcriptional and inflammatory dysregulation.

A critical, yet less understood, upstream trigger for this dysregulation is the repetitive trauma of AVF puncture. Patients typically undergo this procedure twice per HD session, three times per week, amounting to approximately 312 punctures annually. The choice of cannulation device is therefore critical, as it must align with maturation status and minimize vessel injury. Conventional metal needles are associated with several drawbacks, including increased risk of complications during and after maturation, which may lead to session postponement, premature termination, or the need for alternative vascular access (7, 8). Unfortunately, such alternatives are generally inferior to native AVFs and are linked to higher rates of postoperative complications and need for surgical or endovascular reintervention (9). In line with the European Society for Vascular Surgery Guidelines (2018), an optimal cannulation strategy should aim to reduce vascular access damage, procedural complications, and patient pain or anxiety (1).

Plastic cannulas have been used as an alternative to metal needles for decades in many regions (10, 11). In Japan, for instance, where nearly 300,000 patients undergo dialysis weekly, plastic cannulas are the predominant choice, demonstrating high success rates and minimal complications (12, 13). These devices may be particularly advantageous in vulnerable populations, such as patients with diabetes or hypertensive nephropathy, in whom AVF maturation is often delayed and the timing of dialysis initiation is uncertain (14). Additionally, for patients with end-stage renal disease (ESRD) who require emergent dialysis (15), plastic cannulas represent a favorable option due to their reliable performance and reduced complication risk.

Our previous work suggested that plastic cannulas may better preserve long-term AVF patency compared to metal needles (16, 17), particularly in early-cannulation settings. In the present study, we seek to bridge the gap between the established molecular landscape of AVF failure and the clinical observation of device-specific outcomes. While prior research has delineated the transcriptional landscape and identified context-specific biomarkers (3–6), the initiating events, particularly the role of cannulation injury in activating specific inflammatory and complement pathways, remain elusive. Therefore, we aim to elucidate the mechanistic basis by which plastic cannulas confer protection, specifically by testing the hypothesis that they attenuate needle-induced injury by suppressing the activation of these key pathways.

## 1 Methods

### 1.1 Study design and patient enrollment

This retrospective cohort study encompassed all patients with ESRD who underwent primary AVF creation at the 981st Hospital of the PLA, Beidaihe Rehabilitation and Recuperation Center of the PLA, Yichen Hemodialysis Center, and Shenzhen Bao’an District Song Gang People’s Hospital in China, between January 1, 2020, and December 1, 2022. Following a comprehensive review of medical records, 77 patients who underwent their first cannulation between 3 and 4 weeks post-AVF creation were initially identified. The inclusion criteria are outlined in the **Supplementary Material**. After applying these criteria, 64 patients were included: 33 received metal needles, and 31 received plastic cannulas during the first two weeks of cannulation. Between January 1, 2018, and December 31, 2018, 13 of 19 patients refractory to endovascular therapy underwent a second surgical procedure. Among them, five developed recurrent AVF stenosis by December 31, 2019, forming a second small cohort for paired analysis. From one of these five patients, we collected a well-distended outflow vein (WDOV) sample during the second surgery and a high-grade stenotic segment (HGSS) during a third procedure. These two specimens constituted a paired set for microarray analysis. The study received approval from the Ethics Committee of the 981st Hospital of the PLA.

### 1.2 The procedure to follow up

AVF creation and monitoring were performed by four certified HD physicians across the four centers, as detailed in the **Supplementary Materials**.

### 1.3 Definition of AVF failure

AVF failure was defined as a significant dysfunction caused by a hemodynamically significant stenosis, confirmed by duplex ultrasound. Hemodynamically significant stenosis was defined by either imaging or hemodynamic criteria: (1) a luminal diameter reduction of ≥50% on angiography or color Doppler ultrasound; or (2) a reduction in access blood flow (Qa) to < 500 mL/min or a decrease of >25% from baseline. A supporting parameter (e.g., a residual diameter < 2.0 mm) could be used in conjunction with the hemodynamic criteria (18). Failure was clinically manifested by either: (1) acute thrombosis (loss of thrill and bruit); or (2) functional insufficiency, wherein the low Qa resulted in an inability to sustain effective dialysis (e.g., requiring a reduction in prescribed blood flow or leading to persistently high venous pressures), ultimately necessitating an intervention (surgical, endovascular) or a new central venous catheter placement.

### 1.4 Sample collection for microarray

**Figure 1:**
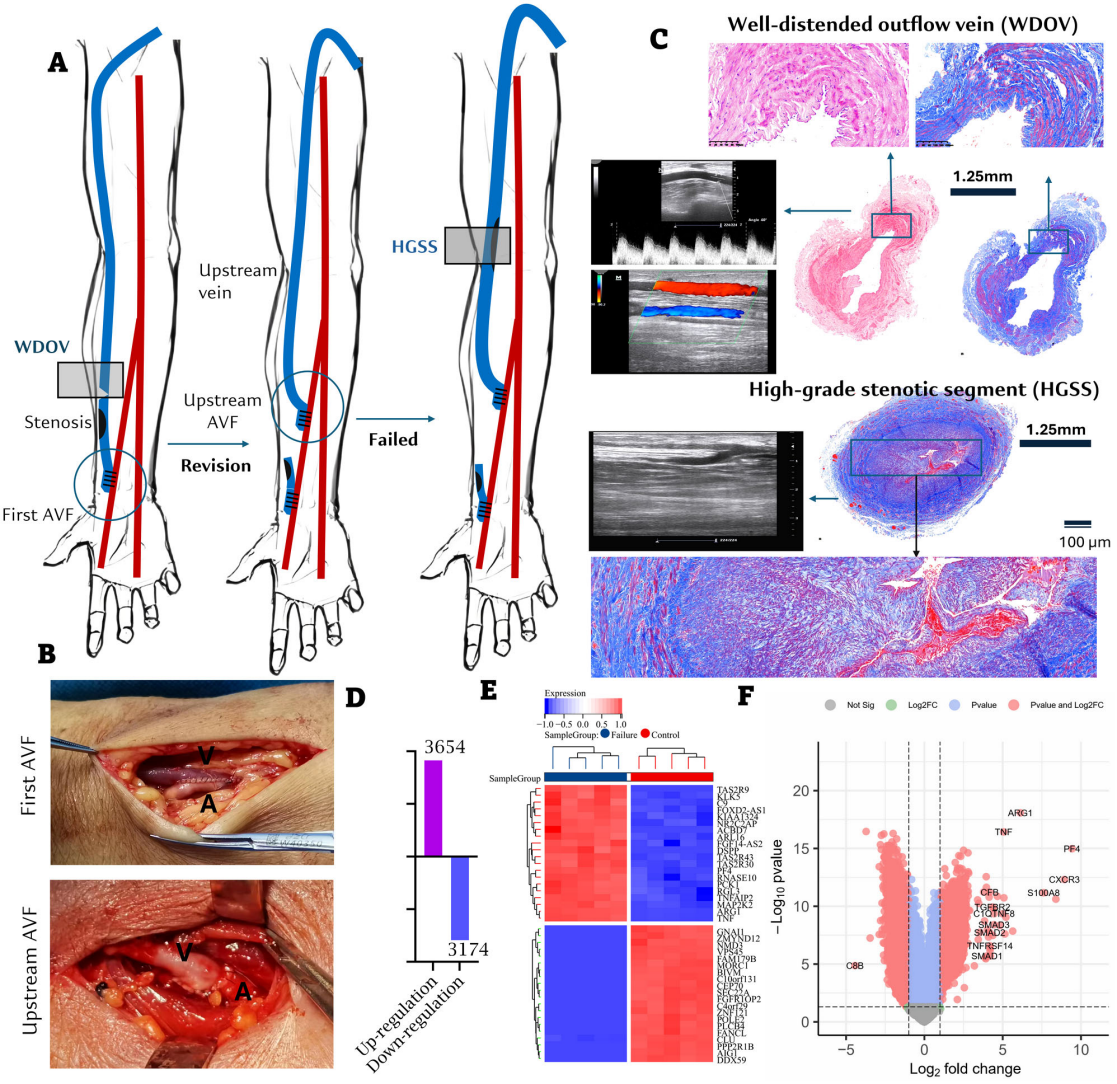
The collection of access samples and microarray. **(A)** The collection of AVF outflow access samples. WDOV, well-distended outflow vein; HGSS, high-grade stenotic segment. **(B)** Initial radio-cephalic (RC) AVF and creation of a new AVF over the upstream of an existing access (Type I secondary fistula). **(C)** Ultrasonic, hemodynamics and macro-pathological images of WDOV and HGSS. WDOV, the diameter of access: 6.4 mm; BF: 962 ml/min; HGSS, shows a long segment of stenosis over cephalic vein fistula and thrombosis formation. PAS and Masson staining show more severe intimal hyperplasia and fibrosis in HGSS compared with WDOV. Bar length: 1.25 mm (macro-pathology) and 100 μm. **(D)** Upregulated and downregulated genes. **(E)** Heatmap of the top 20 DEGs. **(F)** Volcano plot illustrating the expression levels of all DEGs; and the DEGs with Log_2_ (fold changes) >4 were labelled.

From January 1, 2018, to December 31, 2018, 19 patients from four hemodialysis centers experienced AVF failure resistant to endovascular therapy, with causes including extensive fistula aneurysmal changes (n = 4), diffuse stenosis (n = 13), or poor skin condition of the forearm (n = 2). Among these, 13 patients had well-sized and high-quality outflow veins (> 10 cm long, > 6 mm in diameter, < 6 mm under the skin, and BF > 600 mL/min), suitable for the creation of a new AVF upstream. These patients underwent end-to-side anastomosis of the outflow vein to a more proximal inflow artery, with ligation of the failing downstream AVF (**Figures 1A, B**). During the operation, a small segment adjacent to the distal healthy access was excised for the WDOV sample. By December 31, 2019, five of the 13 patients had experienced subsequent AVF failure, leading to excision of the HGSS samples (**Figure 1C**, **Supplementary Figure 1**).

### 1.5 Fluorescence activated cell sorting

Connective tissues from the AVF access were surgically excised, and the access was digested using a calculated volume of enzymatic solution. Sterile scissors were used to mince the tissue into ~1 mm^3^ pieces, which were then treated with pre-warmed collagenase II/DNase I/Dispase II digestion solution (B20332, Sigma, 1 mL/100 mg). The mixture was incubated at 37°C for 1–1.5 hours until the tissue was broken down into fine fragments. The digested tissue solution was passed through a 70 μm strainer, and the cell solution was centrifuged at 300 × g for 5 minutes. The cell pellet was resuspended, and 2 × 10^6^ cells were seeded into a 150 cm^2^ flask pre-coated with fibronectin, with a final volume of 20 mL of endothelial cell medium. The culture was maintained at 37°C in a 5% CO_2_ humidified air incubator until the cells reached 80% confluence. Cells were then centrifuged, and the supernatant was discarded. Antibodies were diluted as follows: APC-conjugated anti-human CD45 (1:100), PerCP-conjugated anti-human CD31 (1:200), FITC-conjugated anti-human CD68 (1:200), PE-conjugated anti-human CD11c (1:200), Alexa 488-conjugated anti-human ARG1 (1:150), Alexa 405-conjugated anti-human TGFβ (1:200), Alexa 594-conjugated anti-human Ly6C (1:100), and Alexa 647-conjugated anti-human αSMA (1:200), each in 100 μL of sterile 5% FBS/PBS solution per tissue sample. An isotype control antibody mixture was similarly prepared to facilitate flow cytometry gating. Cells were collected using fluorescence-activated cell sorting (FACS, Aria III cell sorter, BD Biosciences, San Jose, CA, USA).

### 1.6 Surgical procedure

All radial-cephalic and brachial-cephalic AVFs were created by one team under consistent anesthesia/heparin protocol. A 7–0 polypropylene end-to-side anastomosis was used: a 1 cm arteriotomy at the wrist/forearm for radio-cephalic and a 5 mm arteriotomy at the elbow for brachial-cephalic AVFs.

### 1.7 Sample size calculation

This retrospective study compared AVF failure rates between plastic cannula (*p*_1_ = 0.039) and metal needle (*p*_2_ = 0.351) groups. With 90% power and a 1:1 allocation, the sample size was calculated per Case et al. (19), yielding a requirement of 30 patients per group, which was inflated to 33 to accommodate a 10% loss to follow-up.

### 1.8 The choice of first cannulation time

FCT was defined as the interval from surgery to first successful cannulation, undertaken when the AVF met maturity criteria (blood flow >450 mL/min, diameter >4.5 mm). All procedures used the rope-ladder technique, performed by a senior nurse with 17-G plastic cannulas or metal needles.

### 1.9 Assessment with vascular Doppler ultrasonography

Immediately following anastomosis, we used intraoperative Doppler ultrasonography to measure vessel diameter, peak systolic velocity (PSV), and mean velocity (Vm). We then calculated the blood flow (BF) based on Vm and the vessel’s cross-sectional area.

### 1.10 Microarray

Total RNA was extracted from all tissue specimens. RNA integrity was rigorously assessed using the Agilent Bioanalyzer 2100 system. The RNA Integrity Number (RIN) for each individual sample is provided in **Supplementary Table 1**, with values ranging from 7.5 to 9.1 (mean ± SD: 8.36 ± 0.52). Only samples with a RIN ≥ 7.0 were considered qualified and processed for microarray analysis. The subsequent profiling was performed on RNA from five matched-pair specimens (n=5 pairs), where each pair consisted of a HGSS and a WDOV segment from the same patient. Qualified samples were subjected to Cy3-labeling and microarray hybridization according to the manufacturer’s standard protocols (Agilent). Fluorescent signals were scanned, and the raw expression data were acquired using Feature Extraction software.

### 1.11 Microarray data processing and analysis

Raw data extracted by Feature Extraction software were processed and analyzed using the R software environment (version 4.2.3). The data were first background corrected using the normexp method and normalized between arrays using the quantile method with the limma package (version 3.48.0) (20). Probes were filtered to exclude those with low signal intensity across all arrays. Differential expression analysis between the HGSS and WDOV groups was conducted using an empirical Bayes moderated paired t-test implemented in the limma package. Genes with a Benjamini-Hochberg adjusted *P*-value < 0.05 and an absolute log2 fold change > 1 were considered statistically significant. The microarray data have been deposited in the ArrayExpress database at EMBL-EBI (www.ebi.ac.uk/arrayexpress) under accession number E-MTAB-15287.

### 1.12 Blood samples and assays

Blood samples were collected from the HD vascular access before dialysis and heparin administration, using sodium citrate as the anticoagulant. A panel of complement antigens, including but not limited to CFB, C3a, C5a, and C5b-9, was quantified using commercial ELISA kits.

### 1.13 Immunofluorescence

We performed immunofluorescence on deparaffinized AVF sections using antibodies against CFB and C5b-9, with Alexa Fluor-conjugated secondary antibodies and DAPI counterstain, following the standard protocols. Images were acquired using a confocal microscope and processed with appropriate software.

### 1.14 Western blot

The isolated cells were homogenized in freshly prepared tissue protein extraction reagent (Pierce Bioscience, Rockford, IL, USA). After centrifugation, the supernatants were stored at -80°C. Primary antibodies used included anti-eNOS (ab252439, 1:1000, Abcam) and anti-GAPDH (ab8245, 1:3000, Abcam).

### 1.15 Quantitative real-time PCR

Gene expression was quantified by real-time PCR using SYBR Green Master Mix (Toyobo) on a Rotor-Gene 3000A system (Corbett). Reactions were performed in 20 µL volumes under standard cycling conditions: initial denaturation at 95°C for 1 min, followed by 45 cycles of 95°C for 15 sec and 60°C for 31 sec. All expression levels were normalized to Gapdh and analyzed using the comparative CT method. Primer sequences are provided in **Supplementary Materials**.

### 1.16 *In vitro* experiments

EA.hy 926 endothelial cells (ATCC^®^ CRL-2922™) were cultured in Dulbecco’s Modified Eagle Medium (DMEM, Sigma-Aldrich, # SLM-243-B). The cells were maintained at 37 °C in a humidified atmosphere containing 5% CO_2_. Upon reaching confluence, the cells were detached using 0.25% Trypsin-EDTA and subcultured. All experiments were conducted using cells between passages 3 and 6. Cell proliferation was assessed using both EdU and 5(6)-Carboxyfluorescein N-hydroxysuccinimidyl ester (CFSE) assays on the EA.hy 926 cells, following respective kit protocols and visualized via fluorescence microscopy. Concurrently, nitric oxide (NO) production was measured from culture medium using a nitric oxide analyzer, with concentrations determined against a sodium nitrite standard.

### 1.17 Statistical analysis

Dichotomous variables are expressed as counts (percentages), while continuous variables are presented as means (± standard deviation) or medians (interquartile range). The χ² test was used for comparing dichotomous variables, and t-tests, one-way analysis of variance (ANOVA), and the Wilcoxon rank sum test were employed for continuous variables, as appropriate. Statistical analyses were performed using the Statistical Package for the Social Sciences (version 18.0; SPSS Inc., Chicago, IL) and R software (version 4.2.3; http://www.r-project.org). The bioinformatic and survival analyses were conducted in R using the following key packages: limma (version 3.48.0) for differential expression analysis of microarray data (20); WGCNA (version 1.71) for weighted gene co-expression network analysis (21). clusterProfiler (version 4.0.0) for KEGG, Gene Ontology (GO) enrichment analyses and Gene Set Enrichment Analysis (GSEA) (22). glmnet (version 4.1.3) for LASSO-Cox regression analysis to select prognostic features and calculate the risk score (23). maxstat (version 0.7.25) for determining the optimal cut-off value of the risk score. survival (version 3.2.13) for conducting survival analysis, including the survfit function for Kaplan-Meier estimation and the log-rank test for comparing survival curves. rms (version 6.2.0) for constructing the prognostic nomogram and calculating the Harrell’s concordance index (C-index). pROC (version 1.18.0) for generating receiver operating characteristic (ROC) curves and calculating the areas under the curve (AUCs). Data visualization was aided by the ggplot2 (version 3.3.5) and enrichplot (version 1.12.0) packages. Statistical significance was set at a two-sided *P* < 0.05.

## 2 Results

### 2.1 Identification of hub signal pathway

**Figure 2:**
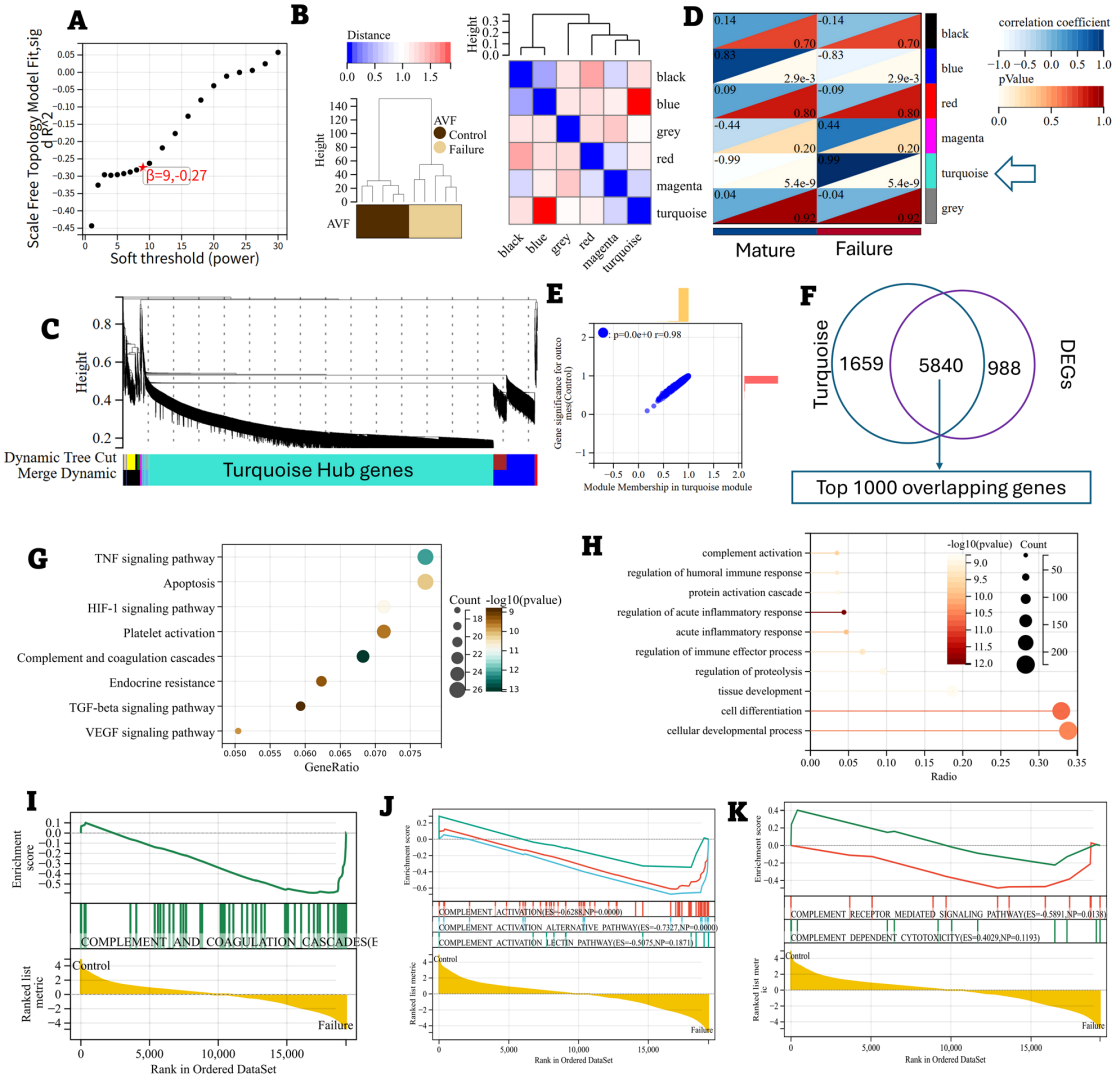
Hub genes associated with AVF failure. **(A–E)** Weighted gene co-expression network analysis (WGCNA) of DEGs. **(A)** Determination of the optimal soft threshold for constructing a scale-free co-expression network. **(B, C)** Cluster dendrogram of all DEGs. **(D)** Correlation between each module and AVF failure **(E)** Correlation between the Turquoise module and AVF failure. **(F)** 5840 overlapping genes between the Turquoise module (n = 7499) and all DEGs (n = 6828). **(G)** KEGG enrichment analysis based on 1000 top overlapping genes. **(H)** Gene ontology (GO) analysis based on 1000 top overlapping genes. **(I–K)** Gene set enrichment analysis plots for the complement pathways.

Microarray data from the ten paired samples (five patients, each contributing a well-distended outflow vein [WDOV] and a high-grade stenotic segment [HGSS]) were analyzed for differentially expressed genes (DEGs) using a paired design. Analysis was performed with the limma package in R, which fits a linear model that accounts for inter-patient variability. We identified a total of 6,828 differentially expressed genes (DEGs) with an adjusted *P*-value < 0.05. Microarray data are available in the ArrayExpress database at EMBL-EBI (www.ebi.ac.uk/arrayexpress) under accession number E-MTAB-15287. Among these DEGs, 3,654 were upregulated, and 3,174 were downregulated (**Figure 1D**). The heatmap for the top 20 DEGs is presented in **Figure 1E**, and the volcano plot for all DEGs is shown in **Figure 1F**. For validation of the microarray findings, the expression levels of select top differentially expressed genes (S100A8, CFB, SMAD2, CXCR3, TNF, and ARG1) were measured by qRT-PCR using the same matched-pair specimens. The qRT-PCR results were consistent with the microarray data, thereby confirming the initial profiling results (**Supplementary Figure 2**). The genes were further processed using the WGCNA package in R software to construct a scale-free co-expression network with a soft thresholding power (β) of 9, which yielded a scale independence of -0.27, indicating good average connectivity (**Figure 2A**). The genes were then clustered into six modules: black (n = 365), blue (n = 994), grey (n = 2), magenta (n = 36), red (n = 58), and turquoise (n = 7,499), with a minimum module size of 30 and a sensitivity value of 3. The cluster dendrogram is shown in **Figure 2B**. The correlation between each module and AVF outcomes was calculated (**Figure 2C**), with the turquoise module demonstrating the most significant positive and negative correlation with AVF failure (r = 0.98, *P* < 0.001, **Figures 2D, E**).

We identified the top 1,000 overlapping genes between the DEGs and the turquoise module, ranked by absolute Log_2_FC, for subsequent functional analysis (**Figure 2F**). KEGG analysis identified the “complement and coagulation cascade” as one of the top activated pathways (**Figure 2G**). Gene Ontology (GO) analysis revealed that these genes were significantly enriched in “complement pathway activation” (**Figure 2H**). Among these, 25 DEGs were found to belong to the complement pathways (**Figure 2I**). Gene Set Enrichment Analysis (GSEA) was conducted to further explore the distribution of different complement pathways in the expression data from both failing AVF and control veins. The “alternative pathway” and “complement receptor-mediated signaling pathway” were notably enriched in the overlapping DEGs, with enrichment scores of 0.7327 and 0.5891, respectively (**Figures 2J, K**).

### 2.2 The alternative complement pathway was notably activated during the first two cannulation weeks

**Figure 3:**
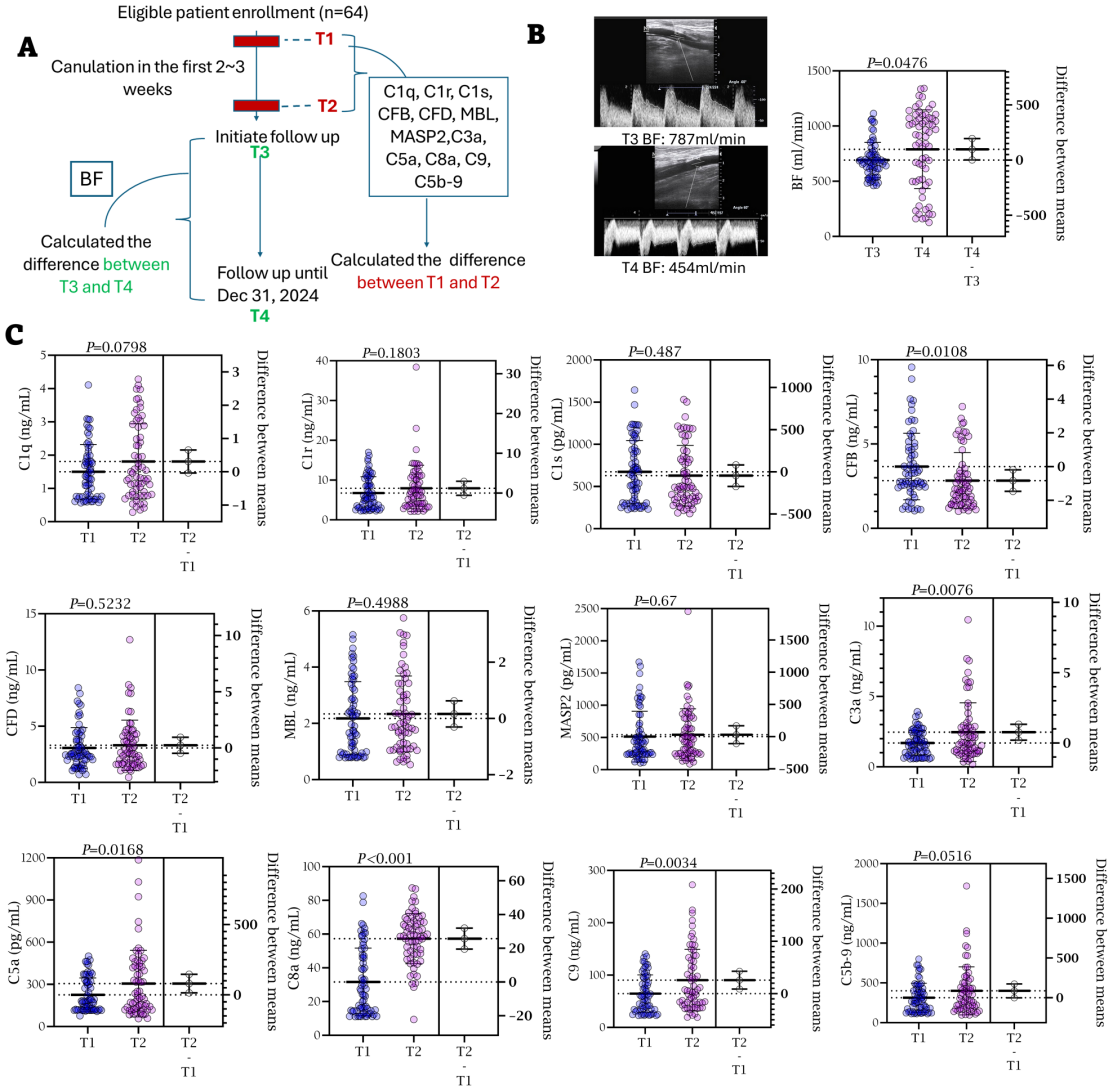
Alternation of blood complement ingredients and blood flow before and after the first two weeks cannulation. **(A)** Patient flowchart. T1, before the first cannulation; T2, after the first two or three-week cannulation; T3, at the baseline of the follow-up; T4, at the endpoint of the follow-up. **(B)** The representative ultrasonic images of one patient with BF decline during the follow-up. BF at T3: 787 ml/min; BF at T4: 454 ml/min. BFs were compared between T3 and T4. **(C)** The changes of complement ingredients during the peri-cannulation period (T1–T2). Depending on the data distribution assessed by normality tests, continuous variables were compared using either Student’s t-tests (reported as mean ± SD) or Wilcoxon rank-sum tests (reported as median with interquartile range [IQR]).

A total of sixty-four patients underwent early first cannulation and were followed for a median of 241 ± 105 days (**Figure 3A**). The study protocol involved distinct, group-specific timelines for biomarker and hemodynamic assessment to precisely evaluate the early effect of cannulation devices. Complement component levels were compared between the pre-cannulation baseline (Timepoint 1, T1) and a post-intervention timepoint (Timepoint 2, T2). The T1–T2 interval was 2 weeks for the metal needle group and 2–3 weeks for the plastic cannula group, reflecting the latter’s initial intervention period. Crucially, the hemodynamic assessment was standardized across all patients. Access blood flow (BF) was measured at a unified timepoint for all patients at the 5th week after first cannulation (Timepoint 3, T3) and again after the full follow-up (Timepoint 4, T4). The values were 692 ± 160 and 790 ± 357 mL/min for T3 and T4, respectively. Changes in BF were calculated asΔ= (T4 - T3)/T3 (**Figure 3B**). This design ensured that the long-term hemodynamic outcomes were compared from an equivalent clinical timepoint, despite the differences in early cannulation strategy.

**Figure 4:**
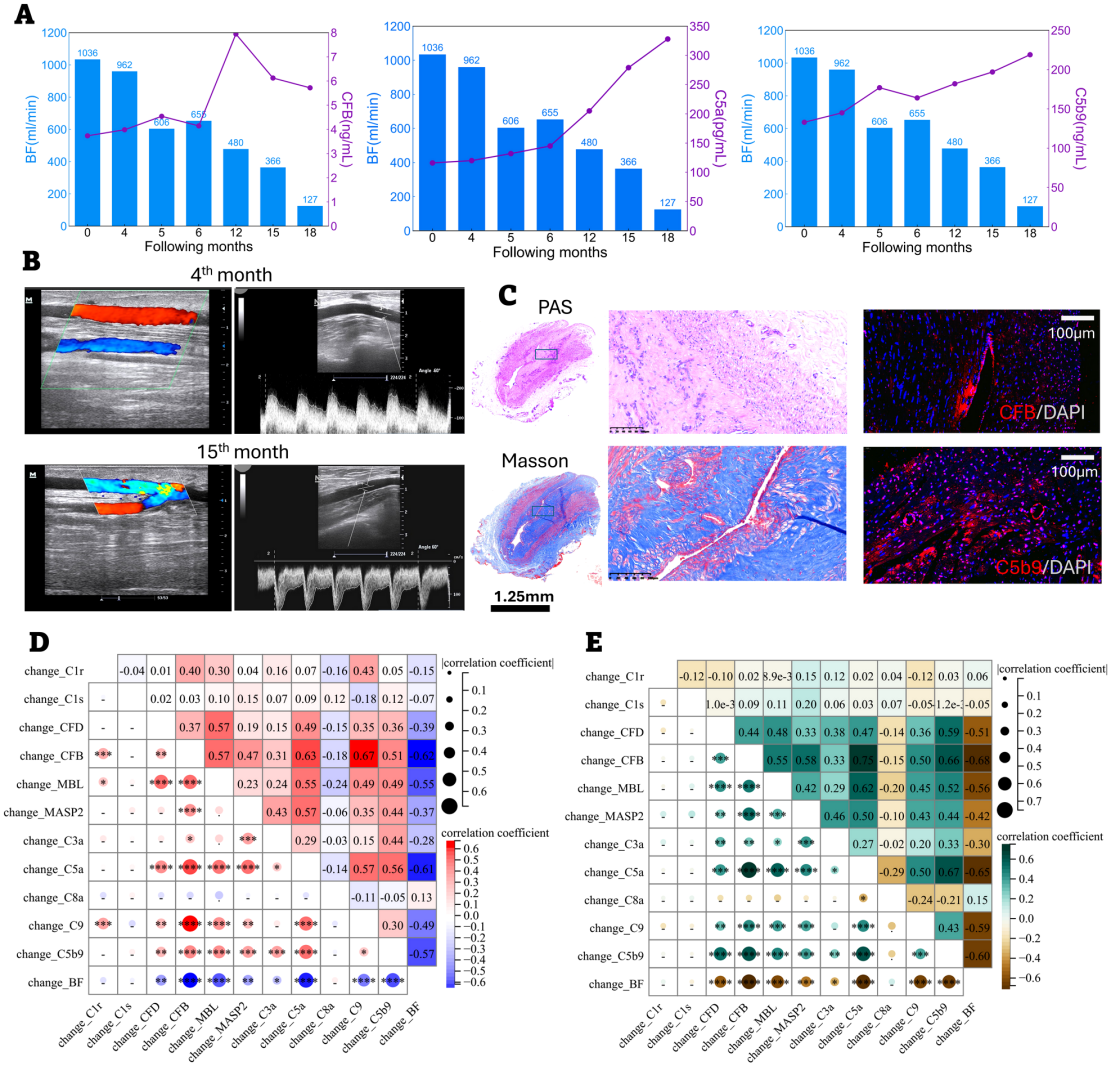
Correlation between the changes of complement ingredients (T1–T2) and the changes of blood flow (BF, T3–T4). **(A–C)** Progressive outflow stenosis with notable alternative complement activation in one patient. **(A)** The increasing CFB, C5a and C5b-9 levels in blood paralleled with the decline of BF. **(B)** The access flow was noted to reduce from 962 ml/min (the 4^th^ month) to 366 ml/min (the 15^th^ month). The patient received balloon angioplasty of AVF in the 15^th^ month. The access flow after fistuloplasty was improved to >500 ml/min. However, his access flow was reduced again to 127 ml/min three months later. In view of rapid recurrence of stenosis after fistuloplasty, surgical revision of the fistula was recommended and the patient agreed with the procedure. **(C)** PAS and Masson staining. The macro-pathological image of intimal hyperplasia, fibrosis and stenosis of access. Bar length: 1.25 mm (macro-pathology) and 100 μm. Immunofluorescent staining. The deposition of CFB and C5b-9 in the intima of failing access. The Pearson **(D)** and Sperman **(E)** correlation matrix relating the T1-to-T2 change in complement ingredients to the T3-to-T4 change in blood flow (BF). *P < 0.05; **P < 0.01; ***P < 0.001; ****P < 0.0001.

Significant differences were observed for CFB, C8, C9, and C5b-9, indicating prominent activation of the alternative pathway during the first two weeks post-cannulation (**Figure 3C**). **Figures 4A, B** illustrate the marked increase in CFB, C5a, and C5b-9 in blood, coinciding with a decline in BF in a 62-year-old male patient, whose access flow decreased from 1036 mL/min to 127 mL/min over 18 months. Balloon angioplasty was performed in the 15th month, resulting in a post-fistuloplasty access flow improvement to > 500 mL/min. However, access flow decreased again to 127 mL/min three months later. Given the rapid recurrence of stenosis, surgical revision of the fistula was recommended. In addition to substantial intimal hyperplasia, immunofluorescence (IF) staining revealed extensive deposition of CFB and C5b-9 in the intima of the failing outflow access (**Figure 4C**). As shown in **Figures 4D, E**, the strongest Pearson and Spearman correlations were found between changes in blood CFB levels and the corresponding changes in BF.

### 2.3 Alternative complement activation accounted for AVF failure

During the follow-up, 13 fistula failures occurred in 64 patients (20.3%). Patient characteristics are detailed in **Supplementary Table 2**. Multivariable analysis confirmed advanced age as a risk factor for AVF failure (adjusted OR = 1.228), while a larger pre-anastomotic cephalic vein diameter (adjusted OR = 0.016) and early plastic cannulation (adjusted OR = 0.019) were potent protective factors. Notably, plastic cannula use was independently associated with the very baseline profiles it benefitted most: older age and smaller venous diameter (**Supplementary Figure 3**).

**Figure 5:**
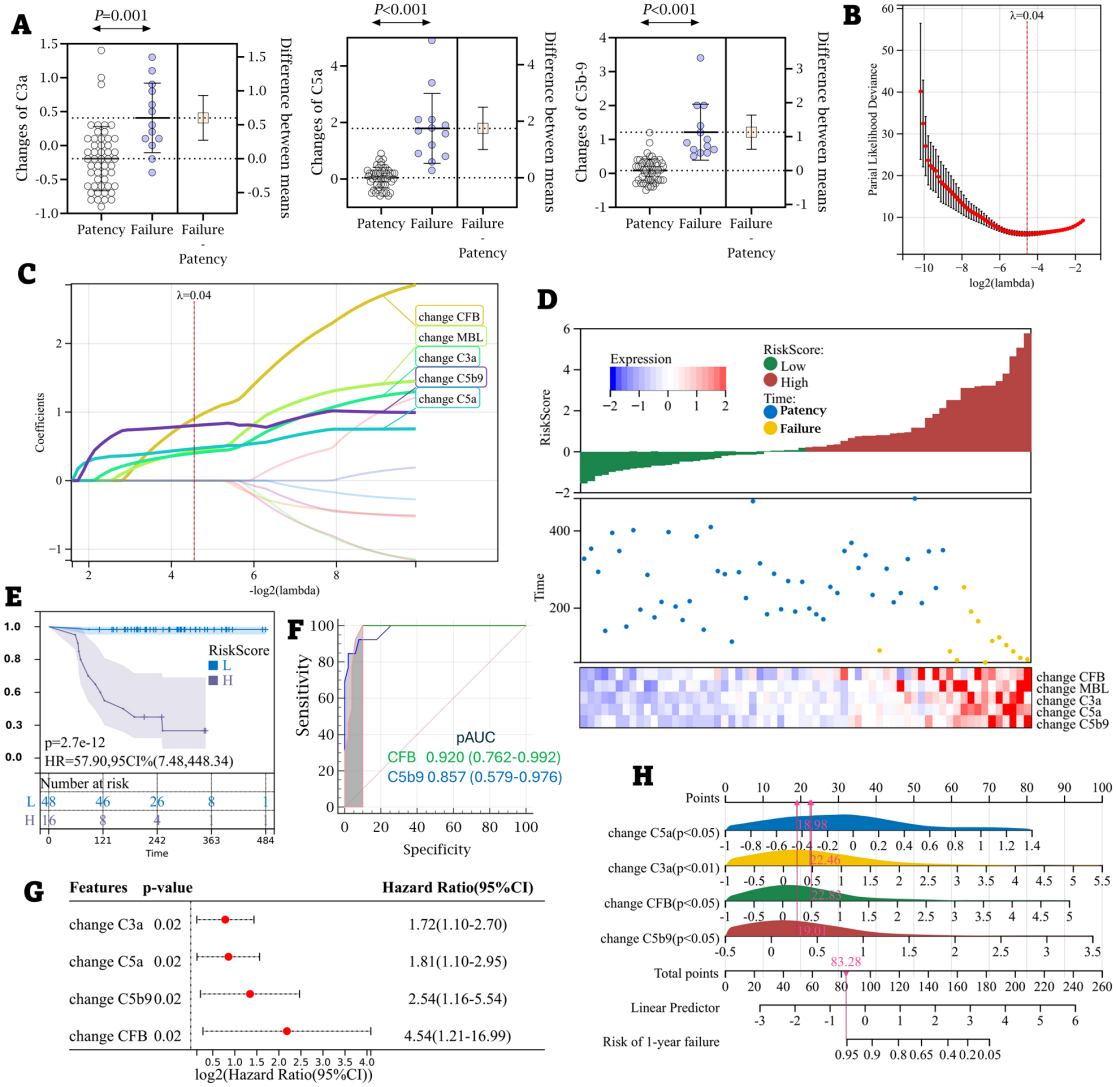
The alternation of CFB during the peri-cannulation period predicted AVF failure. **(A)** The changes of C3a, C5a and C5b-9 were compared between the AVF failure (n=13) and the access patency (n=51). **(B)** The optimal parameter (λ) selection in the LASSO model employed fivefold cross-validation using a minimum criteria approach. The optimal values of λ are represented by dotted vertical lines. Among these values, λ = 0.04 was selected as the optimal choice. **(C)** LASSO coefficient profiles of 12 complement ingredients. The plot was created using a logarithmic scale for the lambda values. A vertical line was added to indicate the lambda value selected through five fold cross-validation. This optimal lambda value led to the identification of five ingredients with nonzero coefficients. **(D)** A composite graph was used to describe the prediction of the changes of CFB, MBL, C3a, C5a and C5b-9 (heatmap) to the risks of AVF failure with consideration of aggregate RSs. **(E)** HR and 95% CI for predicting AVF failure. Red line: high risk (H). Blue line: low risk (L). **(F)** The comparison of areas under ROC curves to predict the risk of AVF failure between the changes of CFB and C5b-9. **(G)** The prediction model with forward multivariate Cox-hazard regression to the risk of AVF failure. **(H)** Nomogram with the changes of CFB, C3a, C5a and C5b-9 predicts the probability of the 1-year AVF failure.

At baseline, complement levels were similar between patients with AVF failure and those with patent access; however, significant differences emerged at T2, with patients experiencing AVF failure showing marked complement activation compared to those with functioning fistulas (**Figure 5A**, **Supplementary Figure 4**, **Supplementary Table 3**). The R package glmnet was employed to integrate survival time, AVF status, and complement expression data for regression analysis using the lasso-Cox method. A five-fold cross-validation determined the optimal model: RiskScore (RS) = 0.905 × ΔCFB + 0.400 × ΔC3a + 0.466 × ΔC5a + 0.431 × ΔC3a + 0.804 × ΔC5b9 (Lambda value = 0.04) (**Figures 5B, C**). Correlation analysis revealed that as RS increased (from left to right on the x-axis, top graph), AVF access patency significantly declined (middle graph). As anticipated, changes in CFB, MBL, C3a, C5a, and C5b-9 were identified as risk factors, with their expression levels rising in line with RS (**Figure 5D**). The R package maxstat was used to calculate the optimal cut-off value for RS, with a minimum sample size greater than 25% and a maximum of less than 75%. The optimal cut-off was set at 1.166, classifying patients into high and low AVF risk groups. The survfit function from the survival package in R analyzed prognostic differences between the two groups. The log-rank test assessed the significance of these differences across sample groups (**Figure 5E**). **Figure 5F** presents the ROC curves for the two markers predicting AVF failure incidence. The estimated areas under the ROC curves (AUCs) were 0.920 and 0.857 for changes in CFB and C5b-9, respectively. Forward Cox proportional hazards regression identified changes in CFB, C3a, C5a, and C5b-9 as independent risk factors for AVF failure (**Figure 5G**). The R package rms was used to integrate AVF survival time, status, and the four complement markers, resulting in the development of a nomogram using the Cox method, which assessed the prognostic value of these complement features in 64 patients (**Figure 5H**).

### 2.4 The appliance of plastic devices suppressed complement activation during the early cannulation

**Figure 6:**
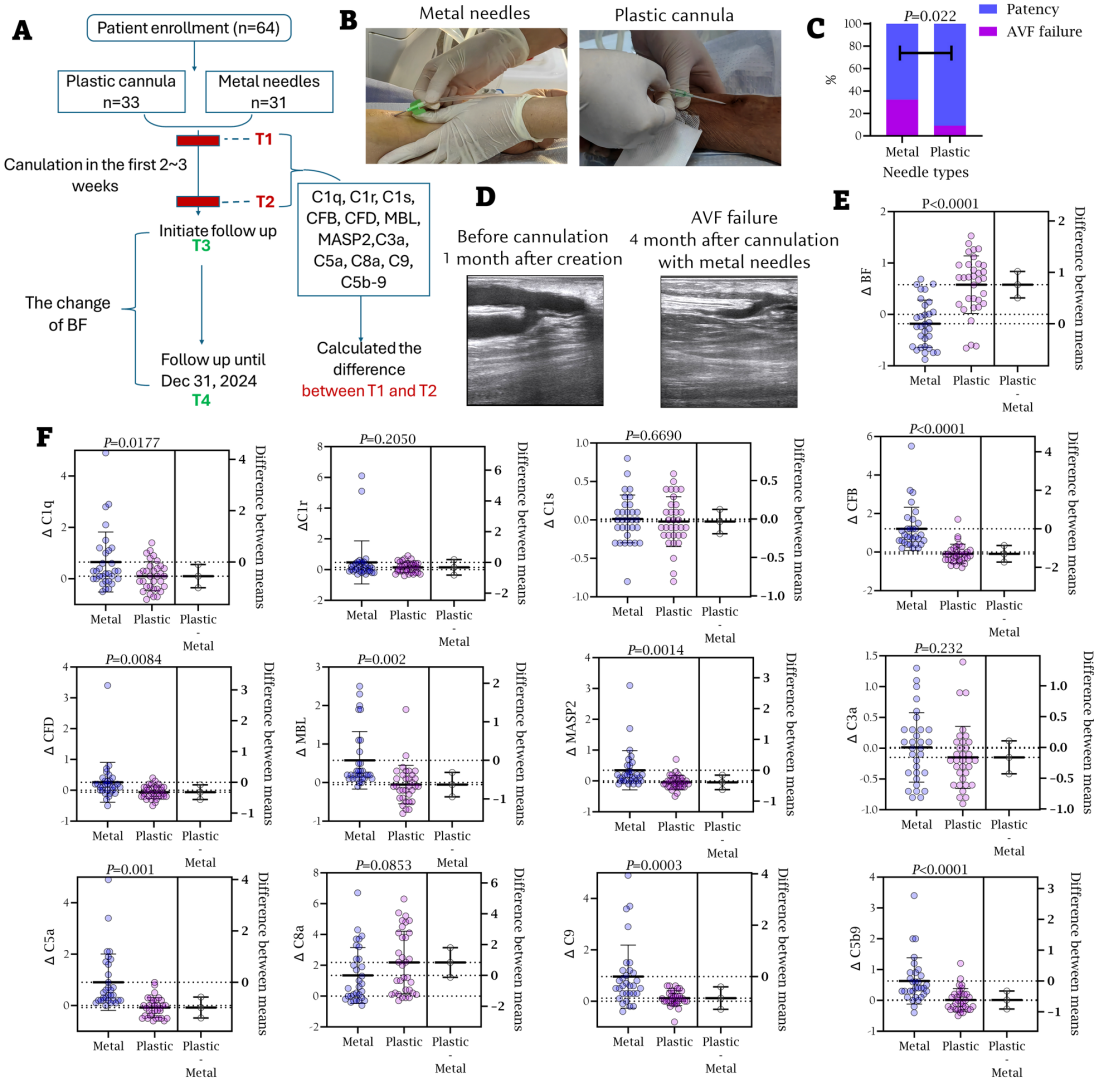
Appliance of plastic cannula suppressed complement activation during the peri-cannulation period. **(A)** Patient flowchart. The patients were divided into two groups according to usage of needle type or plastic cannula during the peri-cannulation period. **(B)** Appliance of metal needles and plastic cannula at the first cannulation. **(C)** The incidence of AVF failure between two groups. **(D)** Representative ultrasonics images of outflow access stenosis after 4-month metal needle usage. The access was unobstructed before the canulation. **(E)** The changes of BF between two groups. **(F)** The changes of complement ingredients in blood between two groups.

Patients were divided into two groups based on the type of needle used during the first 2–3 weeks of cannulation: the metal needle group (n = 31) and the plastic cannula group (n = 33) (**Figures 6A, B**). Patient characteristics for both groups are compared in **Supplementary Table 4**. Among the 33 AVFs in the plastic cannula group, 30 (90.9%) remained functional, compared to 21 of 31 (67.7%) in the metal needle group, with a significant difference (*P* = 0.022, **Figures 6C, D**). In the plastic cannula group, 3 patients experienced fistula failure, two of whom had stenosis in both the anastomosis and juxta-anastomotic region. In contrast, only 1 case of distal stenosis was observed in this group, with no significant stenosis noted at the puncture sites (**Supplementary Figure 5**). Conversely, in the metal needle group, 10 cases of fistula failure occurred. Among these, 8 patients exhibited stenosis in the anastomosis and juxta-anastomotic region, 9 had distal stenosis, and 3 even presented with multiple (more than two) stenoses (**Supplementary Figures 6**, **7**).

As shown in **Figure 6E**, the metal needle group exhibited significantly greater BF decline compared to the plastic cannula group (*P* < 0.001). Complement levels at T1 were similar between the groups, but at T2, the metal needle group showed significantly greater complement activation than the plastic cannula group (**Supplementary Table 5**). Additionally, the changes in CFB, CFD, C5a, C9, and C5b-9 were significantly different between the two groups (**Figure 6F**), indicating that the use of plastic cannulas effectively inhibited the activation of the alternative complement pathways.

### 2.5 The appliance of plastic devices inhibited intimal hyperplasia and inflammatory cell infiltration

**Figure 7:**
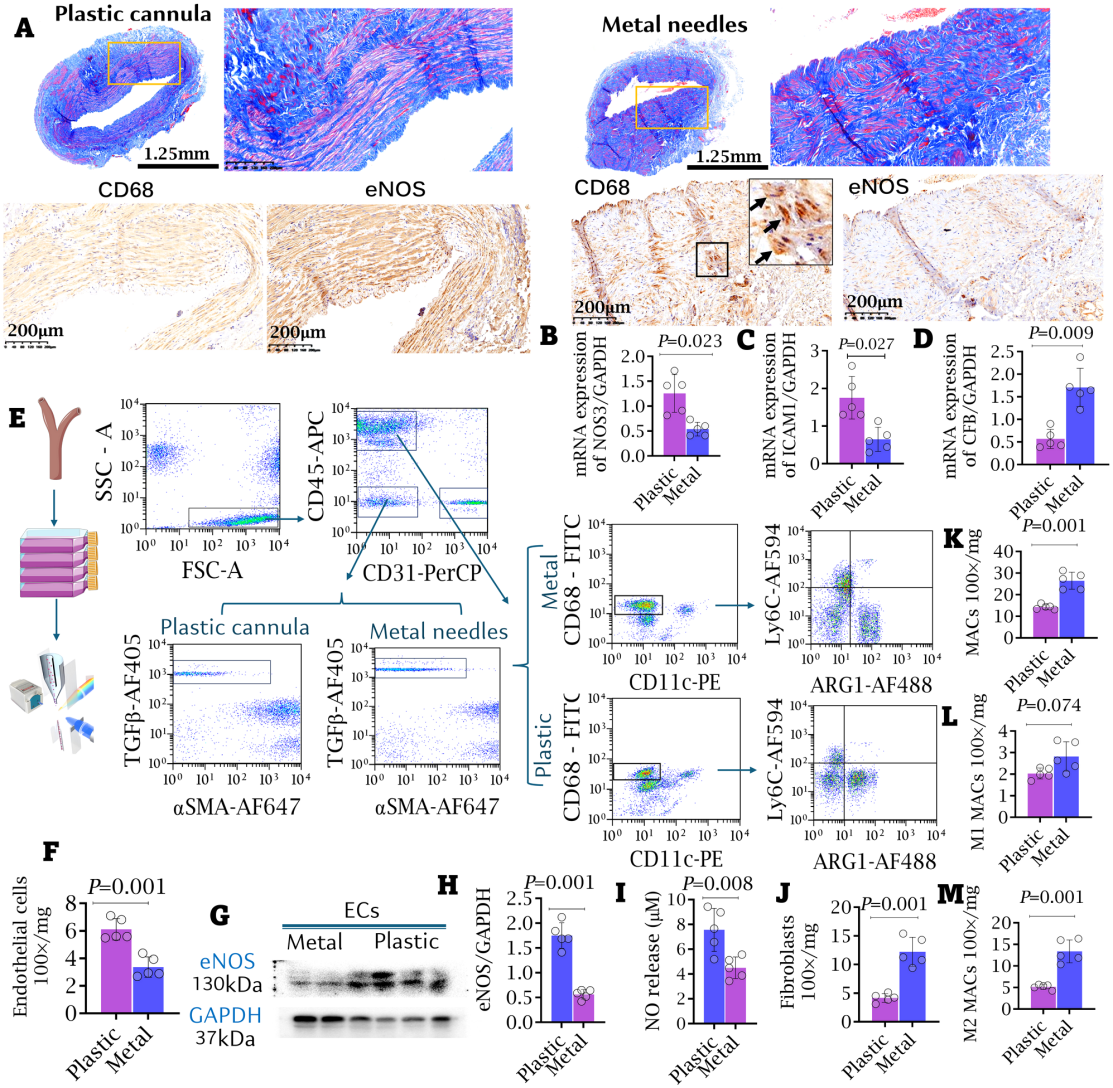
Appliance of plastic cannula alleviated intimal hyperplasia and inflammatory cell infiltrating access. **(A)** The macro-pathological images and immunofluorescent staining against CD68 and eNOS of outflow accesses. More severe intimal hyperplasia and stenosis were observed in the metal needle group. Bar length: 1.25 mm and 200 μm. **(B–D)** mRNA expressions of CFB, ICAM-1 and NOS3 in AVF samples. **(E)** FACS strategy for isolating renal macrophages (MACs), endothelial cells (ECs), and fibroblasts (FIBs) from the access. **(F)** The counts of ECs. **(G, H)** Western blot. The expressions of eNOS in the isolated ECs. **(I)** Nitric oxide (NO) release by isolated ECs. Data were evaluated by NO-analyzer chemiluminescent method. **(J–M)** The counts of FIBs **(J)**, general MACs **(K)**, M1-like **(L)** and M2-like MACs **(M)** in the access were compared between the metal needle group and the plastic cannula group. Data are shown as means ± SD from five independent samples, with experiments performed in triplicate. Analyzed by t-test.

Five patients who received plastic cannulas during early cannulation required open surgical intervention within 3 months of access use. The causes for intervention included thrombus (n = 1), stenosis (n = 1), moderate dialysis-associated steal syndrome (DASS, n = 1), trauma (n = 1), and large hematoma (n = 1). Other samples were obtained from five patients who exclusively used metal needles and experienced failing outflow access. More prominent intimal hyperplasia was observed in the outflow access of patients using metal needles compared to those using plastic cannulas (**Figure 7A**). IHC staining and qRT-PCR both revealed significant upregulation of CFB, coupled with downregulation of ICAM-1 and Nos3 (eNOS) in the intima, paralleling increased macrophage infiltration in the outflow access of patients using metal needles compared to those using plastic cannulas (**Figures 7B–D**). Endothelial cells (ECs), macrophages (MACs), and fibroblasts (FIBs) were isolated from the outflow access using flow cytometry, with macrophages classified into M1- and M2-like phenotypes (**Figure 7E**). EC counts were significantly lower, and eNOS expression was notably suppressed in the metal needle group compared to the plastic cannula group (**Figures 7F–H**). Additionally, nitric oxide (NO) production by ECs from the metal needle group was significantly decreased compared to the plastic cannula group (**Figure 7I**). In contrast, quantification of cell populations revealed significantly higher counts of FIBs and M2-like MACs, rather than M1-like MACs, in the AVF outflow access of patients who always used metal needles compared to those who used plastic cannulas during the early cannulation weeks (**Figures 7J–M**).

### 2.6 The appliances of plastic devices improved EC dysfunction via CFB *in vitro*

**Figure 8:**
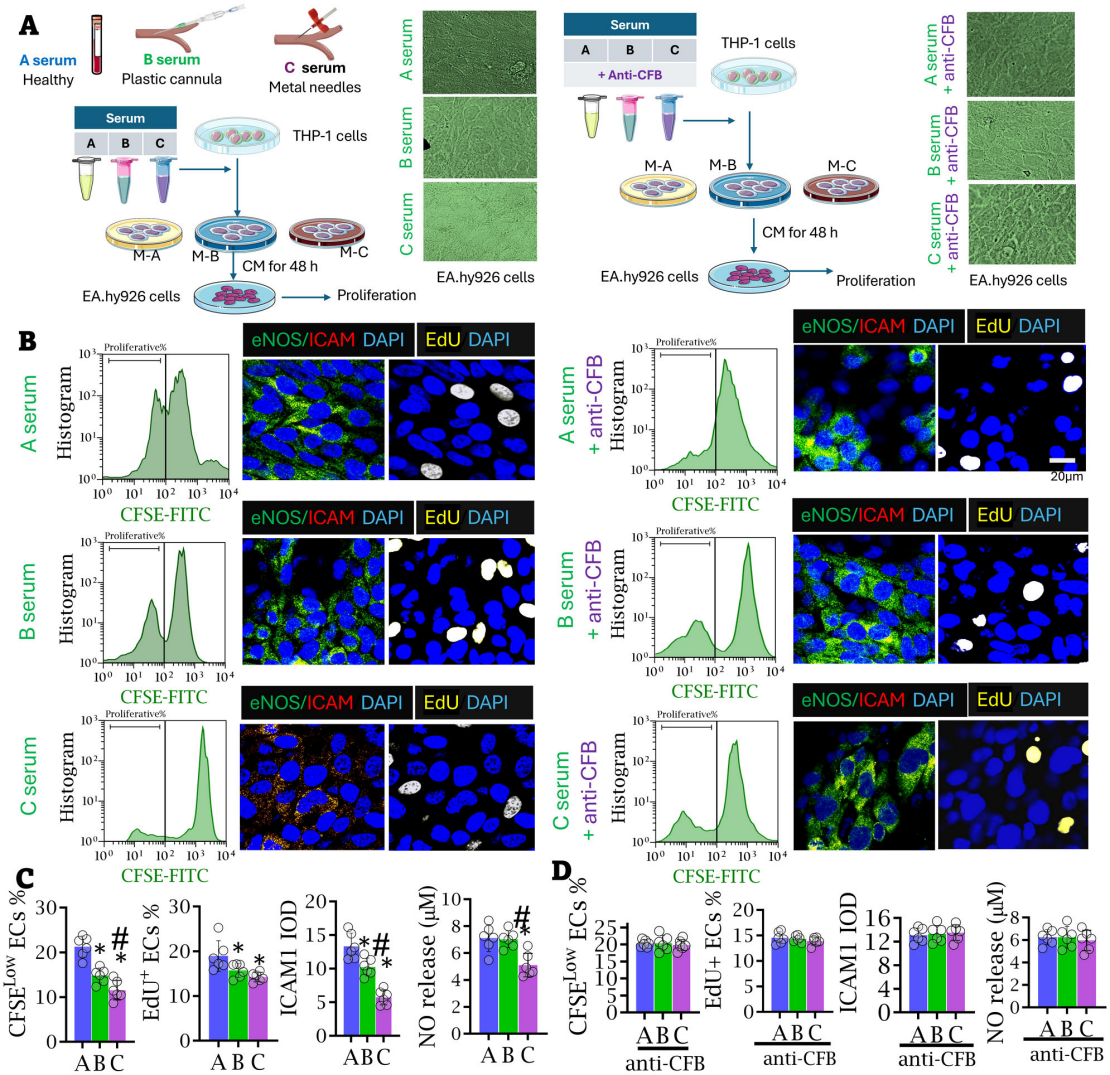
Appliance of plastic cannula inhibited the proliferation of endothelial cells in the intima of access. **(A)** Experimental protocol: THP-1 cells were treated with the serum samples from healthy volunteers (Serum A), patients using metal needles (Serum B), patients using plastic cannula (Serum C) for 24 h, respectively. In another corresponding cell groups, monoclonal anti-CFB antibodies at 1.0 mg/mL were added in the serum samples to neutralize CFB. The supernatant from THP-1 cells was collected 24 hours post-incubation. Then the culture medium (CM) of THP-1 cells was collected and subsequently used to treat EA.hy 926 cells for 48 hours. **(B–D)** The populations of proliferative EA.hy 926 cells were determined by FACS-CFSE and the proportion of EdU-labeled nuclei (yellow). The cytoplasm was stained with eNOS (green) and ICAM-1 (red), and nuclei with DAPI (blue). Images in a group were captured from six independent cell specimens, and the average value of a single cell specimen was achieved from 15 randomized images. Integrated Optical Density values of ICAM-1 expressions in ECs were compared among groups. NO release was measured in the treated EA.hy 926 cells by NO-analyzer chemiluminescent method. Analyzed by one-way ANOVA. *Compared with A serum, *P* < 0.05; ^#^Compared with B serum, *P* < 0.05.

Serum from healthy volunteers (Serum A), from interdialytic patients cannulated with plastic devices (Serum B), and from those using metal needles (Serum C) were collected and incubated with THP-1 cells for 24 hours. Additional groups of THP-1 cells were treated with 1.0 mg/mL anti-CFB antibodies to neutralize CFB in the serum. The culture medium (CM) from THP-1 cells was then collected and used to treat EA.hy 926 cells for 48 hours (**Figure 8A**). As shown in **Figures 8B, C**, CMs from THP-1 cells incubated with Serum B and C significantly inhibited EC proliferation, with the most pronounced effect seen in cells treated with Serum C. ICAM-1 expression was significantly downregulated, and NO production was notably suppressed in the Serum C-treated THP-1 cells compared to the Serum A-treated control group. Interestingly, the anti-proliferative effects on EA.hy 926 cells and the inhibition of NO production were not observed in any serum samples when CFB was neutralized by monoclonal anti-CFB antibodies (**Figure 8D**).

## 3 Discussion

This study demonstrates that early cannulation with metal needles initiates a pathological cascade by exacerbating alternative complement pathway activation. The upregulation of CFB impedes endothelial cell proliferation and promotes neointimal hyperplasia. Conversely, plastic cannulas markedly suppress this detrimental complement activation, thereby preserving EC function and reducing wall thickening, revealing a novel mechanism underlying their clinical superiority.

A major factor specific to AVF failure is inflammation stemming from both surgical creation and repetitive cannulation. The hemodynamic impact of the needle type is a critical initiating event. The design of plastic cannulas, with their side holes, reduces jet velocity from the central bore, thereby minimizing adverse wall shear stress on the venous endothelium (24). In contrast, metal needles generate a disturbed, low-speed jet pattern that is a recognized trigger for intimal hyperplasia (25, 26).

This biomechanical insult directly translates into dysfunctional endothelial phenotypes. While laminar, high wall shear stress (WSS) promotes a quiescent endothelium and NO release, the oscillatory, low WSS induced by metal needles suppresses NO synthesis and upregulates pro-inflammatory genes in ECs (27, 28). Our findings confirm this: ECs from the metal needle group exhibited significantly reduced proliferation, lower NO production, and downregulated eNOS expression compared to those from the plastic cannula group. Further, serum from metal needle patients potently inhibited EC proliferation and NO synthesis *in vitro*.

The injured endothelium subsequently serves as a pro-inflammatory platform, initiating a self-reinforcing cycle between platelet and complement activation—two of the most significantly upregulated pathways in failing accesses. Platelets adhere to the endothelium, releasing cytokines (MCP-1, VCAM-1, ICAM-1, IL-1β, TNF-α). This process is bidirectionally linked to complement, where factors like MBL activate platelets (29), and platelet-derived proteins, in turn, modulate classical complement activation (30). This synergistic interplay amplifies endothelial dysfunction and leukocyte recruitment. Platelets also promote endothelial microparticle release via P-selectin and Mac-1 (31), which further inhibits NO and exacerbates the vicious cycle of hyperplasia (32, 33).

The inflammatory cascade culminates in a maladaptive remodeling of the immune microenvironment (34), decisively driven by monocytes/macrophages (35). We found a significant increase in arginase-1-dominant (M2-like) macrophages in stenotic specimens, underscoring their detrimental role (36, 37). This M2 polarization is mechanistically linked to complement activation, as CFB—also upregulated in stenotic tissues—significantly increases arginase-1 expression (38). Arginase-1, in turn, stimulates fibroblast and vascular smooth muscle cell proliferation (38, 39), directly driving the vessel wall thickening and neointimal formation that characterize access stenosis (40, 41). This defines a concrete pathological pathway: CFB-containing serum inhibited EC proliferation via M2-like monocytes, an effect reversed by CFB neutralization. Crucially, metal needle use enhanced arginase-1+ macrophage infiltration *in vivo*, positioning M2 polarization as the critical link between puncture injury and structural remodeling.

This integrated pathway—from jet impingement to sustained inflammation and immune-mediated remodeling—is robustly supported by our clinical observations. Stenosis in plastic cannula patients was predominantly localized to the anastomosis, with almost no distal involvement. In stark contrast, metal needle patients exhibited widespread stenosis encompassing both the perianastomotic area and the distal outflow vein, highlighting the critical role of needle-induced injury.

Our model also elucidates the predominant localization of hyperplasia at the anastomosis. Although puncture is intermittent, the resultant injury ignites a systemic humoral response, including persistent overproduction of complement components like CFB. These components accumulate at the anastomotic site during interdialytic intervals, fostering a pro-fibrotic niche rich in M2 macrophages that drives wall thickening, thereby explaining how a localized puncture injury converges into a systemic response at the anatomically susceptible anastomosis.

## 4 Limitations and future directions

This study has several limitations. Its retrospective nature resulted in a relatively small sample size despite our matching efforts, with potential for inherent selection bias. Furthermore, our exploration of the mechanisms linking cannulation injury to stenosis, while revealing a key CFB-M2 pathway, is not exhaustive. The pathophysiology likely involves other critical mechanisms, such as sustained platelet activation and its complex interplay with inflammatory networks. Finally, this study cannot fully delineate the causal relationships between complement activation, platelet activation, and endothelial injury, which may form a complex positive-feedback loop. Future studies with larger, prospective cohorts and targeted interventional designs are warranted to verify these causal links and explore additional pathological mechanisms.

In summary, this work delineates a coherent pathological axis—from the physical trauma of cannulation, through the hemodynamic perturbation of WSS, to the molecular activation of complement and platelets, and finally to the cellular execution of M2 macrophage-driven fibrotic remodeling. It establishes the choice of cannulation device not merely as a procedural detail, but as a decisive factor influencing the fundamental biological fate of the vascular access.

## Data Availability

The data that support the findings are included within the manuscript and the Supplemental materials. The microarray data have been deposited in the ArrayExpress database at EMBL-EBI (www.ebi.ac.uk/arrayexpress) under accession number EMTAB-15287.
