# Supplementary material for: Plastic cannulas mitigate arteriovenous fistula stenosis by suppressing the CFB-mediated inflammatory cascade
Source: Front Immunol. 2025 Dec 8;16:1715417. doi: 10.3389/fimmu.2025.1715417 (PMC12719519; doi:10.3389/fimmu.2025.1715417)
Supplement: Supplementary file 1 [file Presentation1.pdf]

Supplementary Tables

Table 1. RNA Integrity Number (RIN) Values for Matched-Pair Specimens Used in Microarray Analysis

Patient Pair ID	Sample type	RIN value
Pair 1	HGSS	8.2
	MDOV	8.5
Pair 2	HGSS	7.5
	MDOV	7.9
Pair 3	HGSS	9.1
	MDOV	8.8
Pair 4	HGSS	7.8
	MDOV	8.1
Pair 5	HGSS	8.7
	MDOV	8.9

Range: 7.5−9.1
Mean±SD: 8.36±0.52

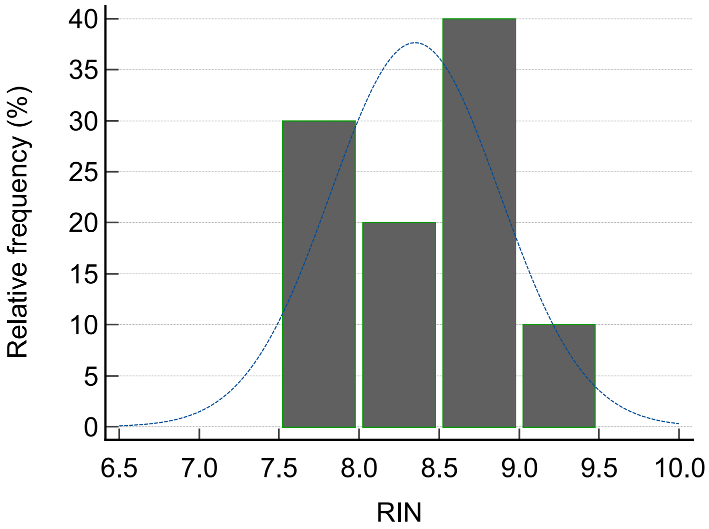

HGSS, high-grade stenotic segment; WDOV, well-distended outflow vein.

Table 2. Patient characteristics.

Demographic and clinical data		AVF access information	
Age (years)	56.2 (15.2)	CVC, n (%)	46 (71.9)
Male %	43 (67.2)	Kt/V	1.11 (0.13)
Diabetes %	26 (40.6)	HD hours/session	4.05 (0.15)
Weight %	6.69 (1.09)	CVD (operation) (mm)	2.25 (0.46)
SBP (mmHg)	127 (26)	CVD (3rd week) (mm)	4.82 (0.32)
DBP (mmHg)	78 (19)	BF (TP3) (mL/min)	692 (160)
		BF (TP4) (mL/min)	790 (357)
Etiology of ESRD			
Hypertension	10 (15.6)	Dialyzer types	
Diabetes mellitus	19 (29.7)	Low-flux	36 (56.3)
Glomerulonephritis	23 (35.9)	Medium-flux	17 (26.6)
ADPKD	5 (7.8)	High-flux	11 (17.2)
Others	7 (10.9)		
		Anti-platelet agent	
Biochemical data		Aspirin	25 (39.1)
Cr (mg/dL)	7.39 (1.37)	Clopidogrel	2 (3.1)
eGFR (mL/1.72m^2.min)	8.06 (2.36)		
ALB (g/L)	33.1 (3.4)	Anticoagulation	
Ca (mmol/L)	2.06 (0.10)	UFH	38 (59.4)
P (mmol/L)	2.45 (0.09)	LMWH	25 (39.1)
iPTH (pg/mL)	317 (190)		
FGF-23 (pg/mL)	442 (175)		
TC (mg/dL)	150.8 (6.0)		
TG (mg/dL)	123.0 (5.5)		
Hb (g/L)	86.4 (7.3)		
PLT (10^9/L)	168 (53)		

Abbreviation: SBP, systolic blood pressure; DBP, diastolic blood pressure; ADPKD, autosomal dominant polycystic kidney disease; Cr, creatinine; ALB, albumin; P, phosphorus; iPTH, intact parathyroid hormone; FGF-23, fibroblast growth factor-23; TG, total cholesterol; TC, triglyceride; Hb, hemoglobin; PLT, platelet; CVC, central venous catheter; CVD, cephalic vein diameter; BF, blood flow; TP, timepoint; UFH, unfraction heparin; LMWH, low molecular weight heparin.

Table 3. Comparison of alternative complement ingredient levels between Timepoint 1 and 2.

	Timepoint 1			Timepoint 2		
	AVF failure n=13	Access patency n=51	P value	AVF failure n=13	Access patency n=51	P value
CFD (ng/mL)	2.73 (1.67)	3.16 (1.85)	0.453	4.38 (3.43)	3.03 (1.76)	0.049
CFB (ng/mL)	4.43 (1.74)	3.47 (2.00)	0.118	5.61 (0.96)	2.12 (0.85)	<0.001
C3a (ng/mL)	2.06 (1.06)	1.60 (0.83)	0.096	5.53 (2.56)	1.68 (0.93)	<0.001
C5a (pg/mL)	192 (106)	234 (123)	0.267	541 (345)	256 (155)	<0.001
C8a (ng/mL)	30.0 (17.0)	32.1 (20.9)	0.735	49.8 (18.5)	59.2 (13.3)	0.040
C9 (ng/mL)	62.9 (36.3)	65.0 (36.2)	0.849	129 (72)	80 (51)	0.007
C5b-9 (ng/mL)	289 (183)	320 (183)	0.586	629 (431)	341 (230)	0.002

Table 4. The characteristics of patients using plastic cannula and metal needles.

	Plastic canula (n=33)	Metal needle (n=31)	
	Demographic and clinical information		
Age (years)	59.3 (14.2)	52.9 (15.7)	0.093
Male %	24 (72.7)	19 (61.3)	0.240
Diabetes %	17 (51.5)	9 (29)	0.057
Weight %	6.6 (1.1)	6.7 (1.1)	0.778
SBP (mmHg)	128 (15)	124 (32)	0.521
DBP (mmHg)	80 (10)	75 (25)	0.267
	Biochemical examination		
ALB (g/L)	32.7 (3.3)	35.5 (3.5)	0.040
Ca (mmol/L)	2.05 (0.08)	2.07 (0.10)	0.319
P (mmol/L)	2.45 (0.09)	2.44 (0.09)	0.539
iPTH (pg/mL)	323 (199)	310 (181)	0.775
FGF-23 (pg/mL)	437 (170)	447 (182)	0.812
Hb (g/L)	83.4 (6.0)	89.7 (7.3)	0.001
	AVF access information		
CVC before AVF, n (%)	22 (66.7)	24 (77.4)	0.249
Kt/V	1.08 (0.11)	1.15 (0.15)	0.016
HD hours/session	4.08 (0.18)	4.01 (0.09)	0.105
CVD pre-operation (mm)	2.14 (0.43)	2.36 (0.48)	0.069
CVD 3rd week (mm)	4.81 (0.39)	4.84 (0.23)	0.730
BF TP3 (mL/min)	639 (141)	748 (163)	0.006
BF TP4 (mL/min)	969 (284)	599 (329)	<0.001

Abbreviation: SBP, systolic blood pressure; DBP, diastolic blood pressure; Cr, creatinine; ALB, albumin; P, phosphorus; iPTH, intact parathyroid hormone; FGF-23, fibroblast growth factor-23; Hb, hemoglobin; CVC, central venous catheter; CVD, cephalic vein diameter; BF, blood flow; TP, timepoint.

Table 5. Comparison of alternative complement ingredient levels between Timepoint 1 and 2.

	Timepoint 1			Timepoint 2		
	Plastic cannula n=33	Metal needle n=31	P value	Plastic cannula n=33	Metal needle n=31	P value
CFD (ng/mL)	2.97 (1.94)	3.19 (1.69)	0.638	2.77 (1.87)	3.87 (2.47)	0.050
CFB (ng/mL)	3.64 (1.88)	3.70 (2.10)	0.905	2.21 (1.09)	3.38 (2.07)	0.015
C3a (ng/mL)	1.64 (0.88)	1.74 (0.92)	0.651	1.30 (0.67)	3.70 (2.39)	<0.001
C5a (pg/mL)	218 (119)	233 (122)	0.617	201 (140)	269 (49)	<0.001
C8a (ng/mL)	31.4 (20.8)	31.9 (19.6)	0.927	49.4 (16.1)	64.8 (8.7)	<0.001
C9 (ng/mL)	56.6 (33.9)	73.1 (36.6)	0.066	60.7 (36.0)	121.9 (62.5)	<0.001
C5b-9 (ng/mL)	286 (172)	343 (191)	0.208	259 (122)	551 (361)	<0.001

Supplementary Figures

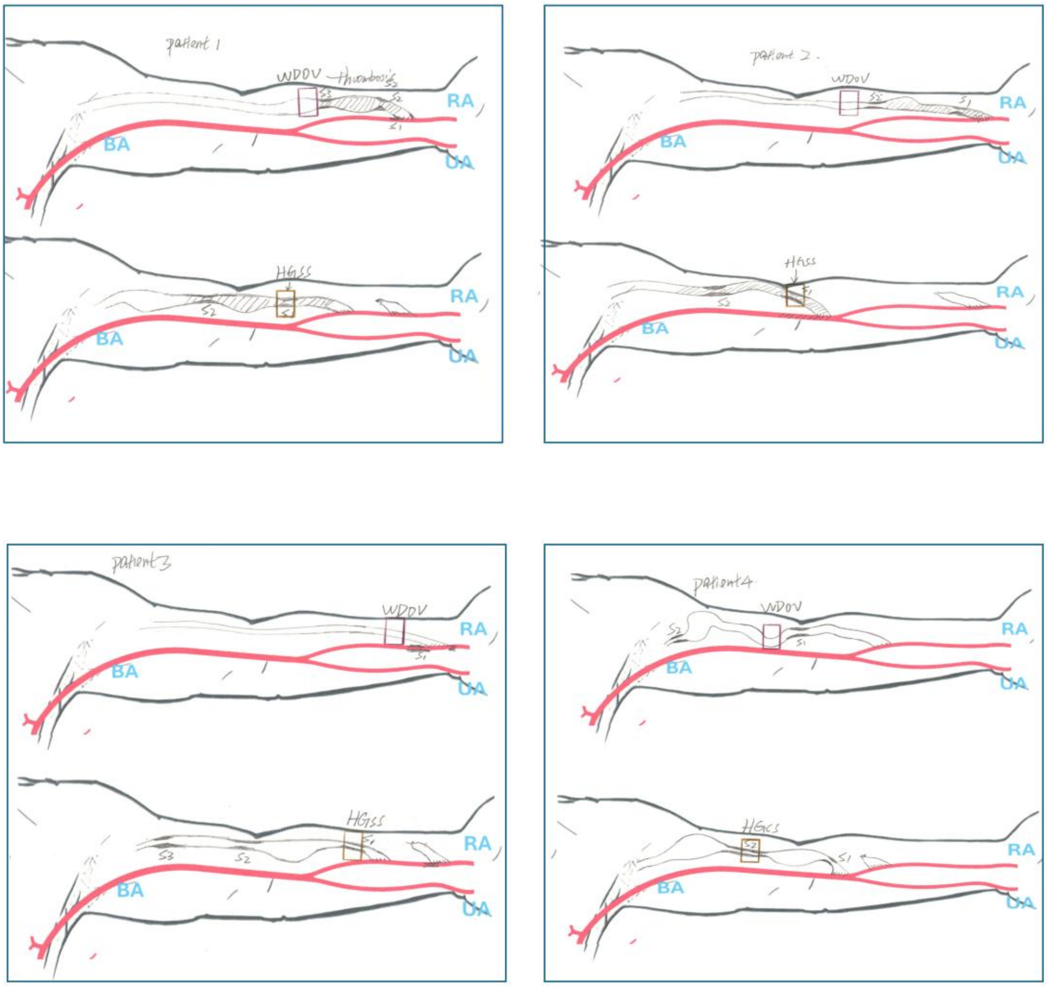

Figure 1. Anatomical sites of the paired tissue samples. Schematic shows the collection of well-distended outflow vein (WDOV) and high-grade stenotic segment (HGSS) samples from four patients for microarray analysis.

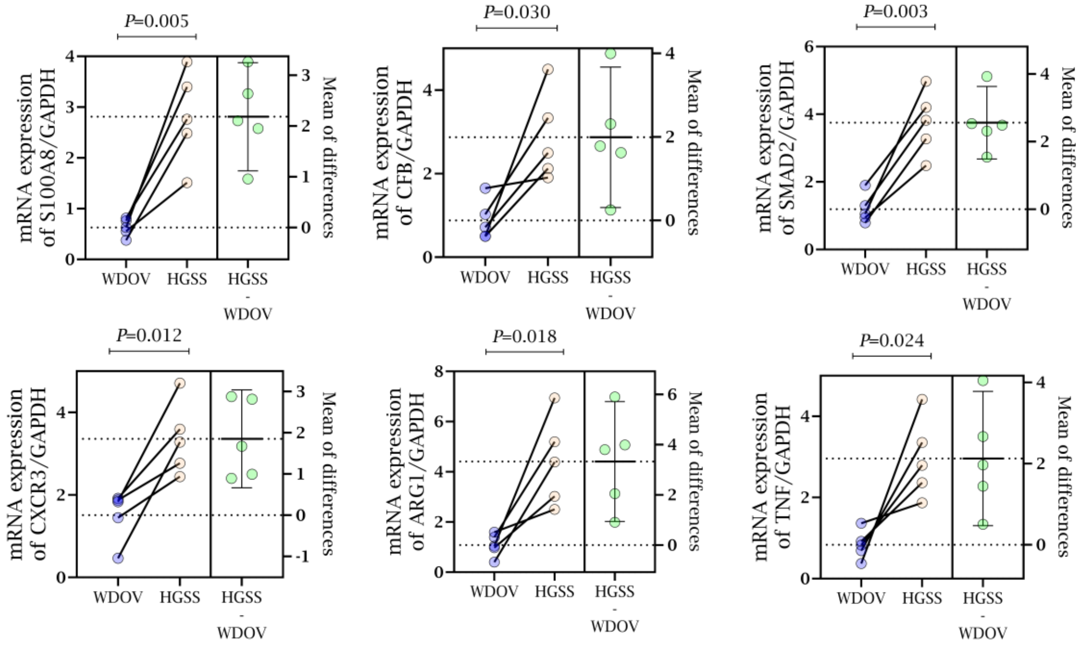

Figure 2. qRT-PCR analysis of S100A8, CFB, SMAD2, CXCR3, TNF, and ARG1 expression in matched-pair specimens (n=5). Statistical significance was determined by a paired *t*-test.

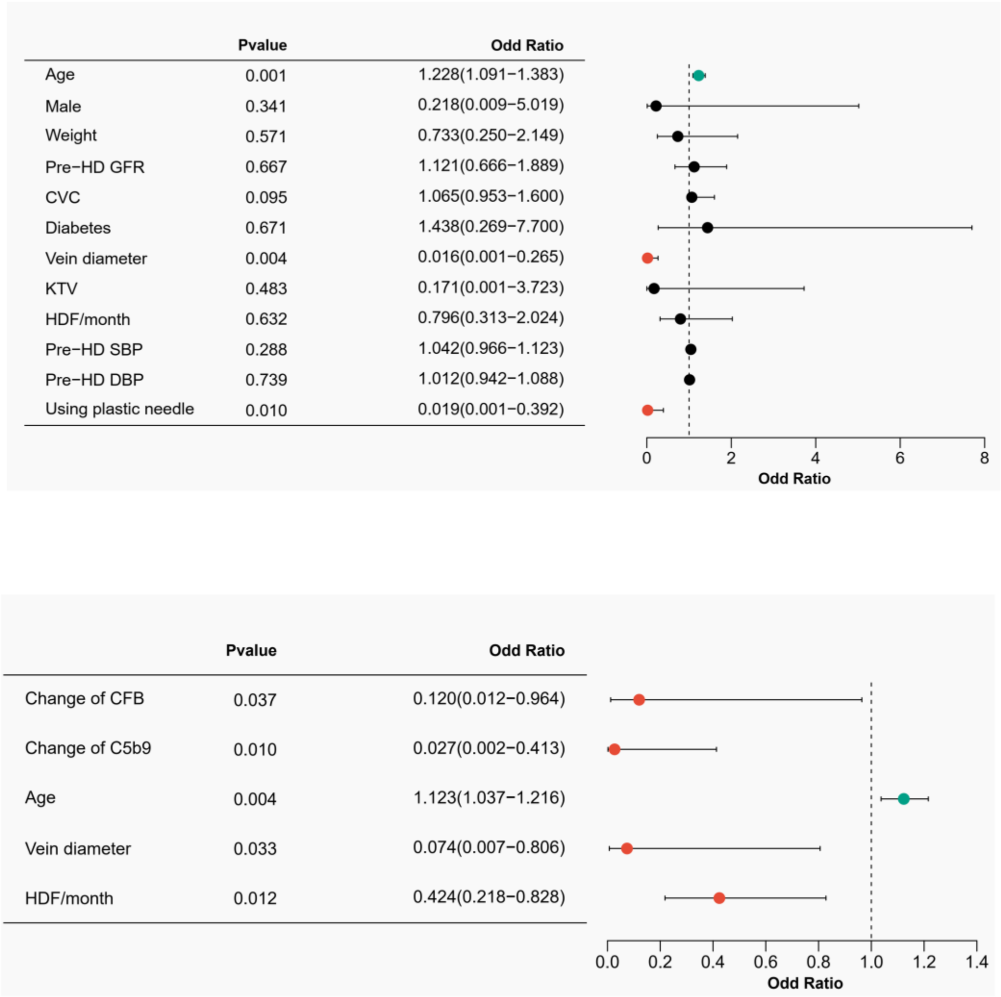

Figure 3. Association of clinical baseline characteristics with AVF failure and plastic cannula usage, as determined by forward stepwise logistic regression analysis.

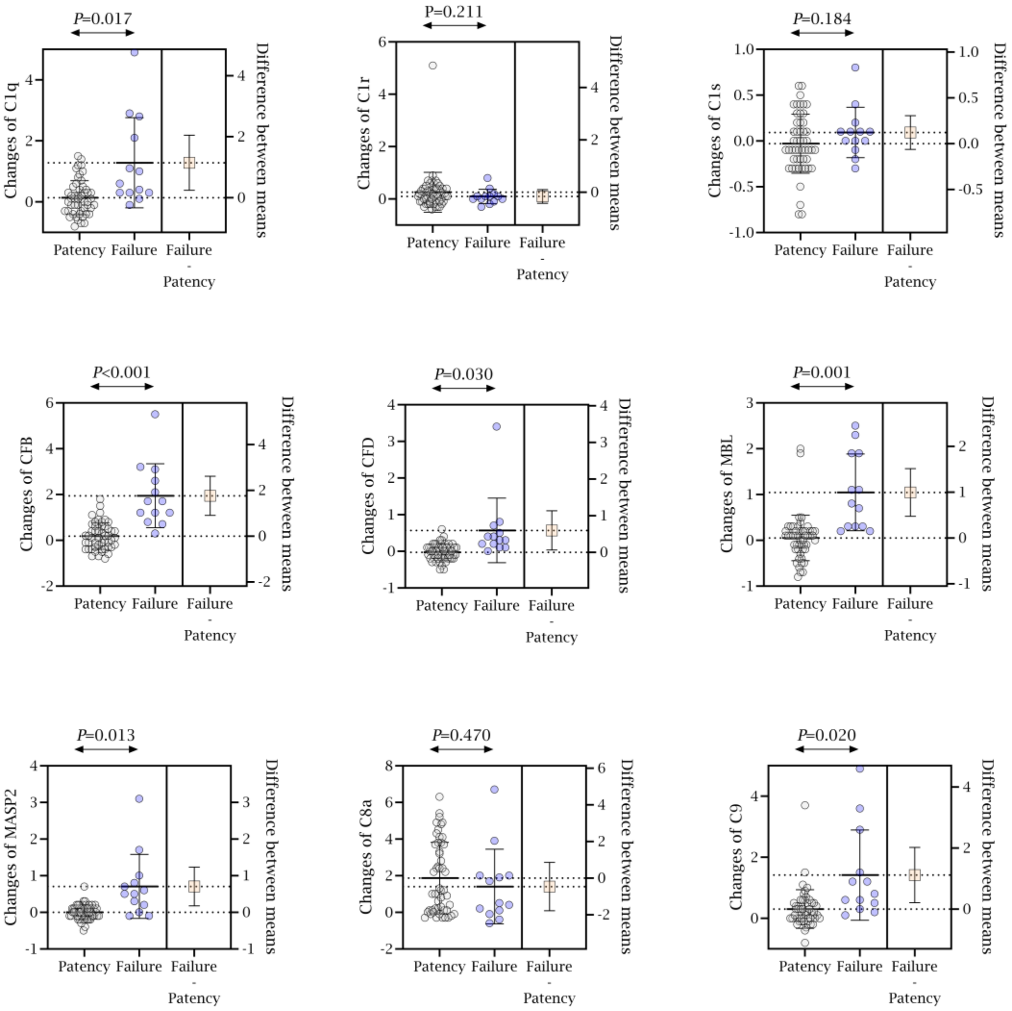

Figure 4. Changes in complement components in patients with AVF failure (n=13) versus those with patency (n=51).

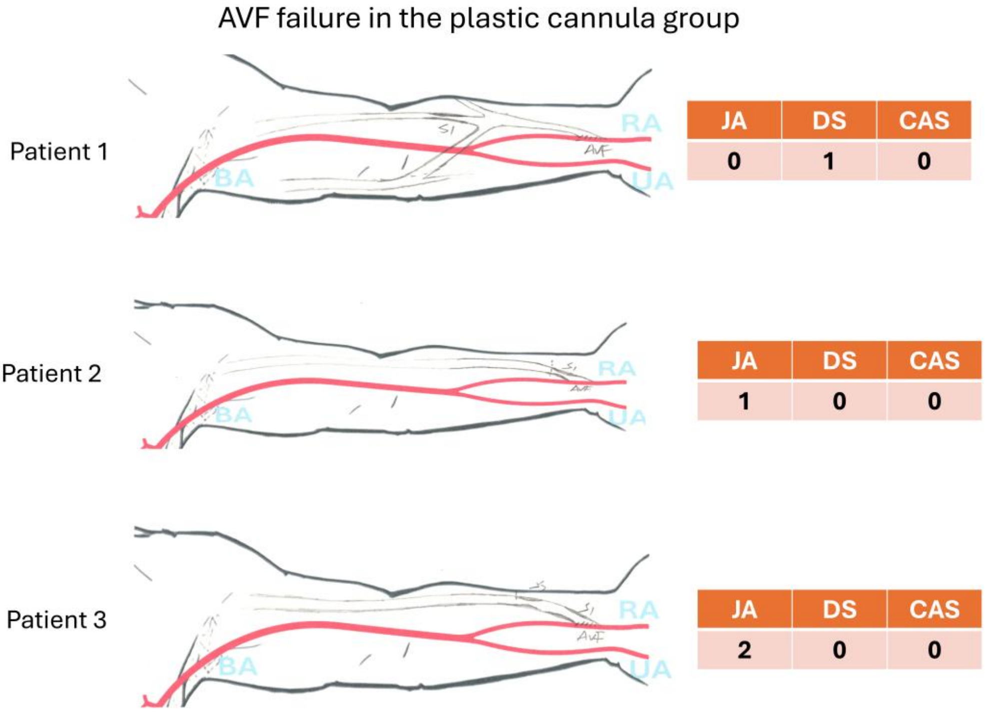

Figure 5. Anatomical locations of stenosis in three patients from the plastic cannula group. Schematic shows stenotic sites (S, numbered sequentially) in the juxta-anastomotic (JA) region, distal outflow (DS), and cannulation area stenosis (CAS).

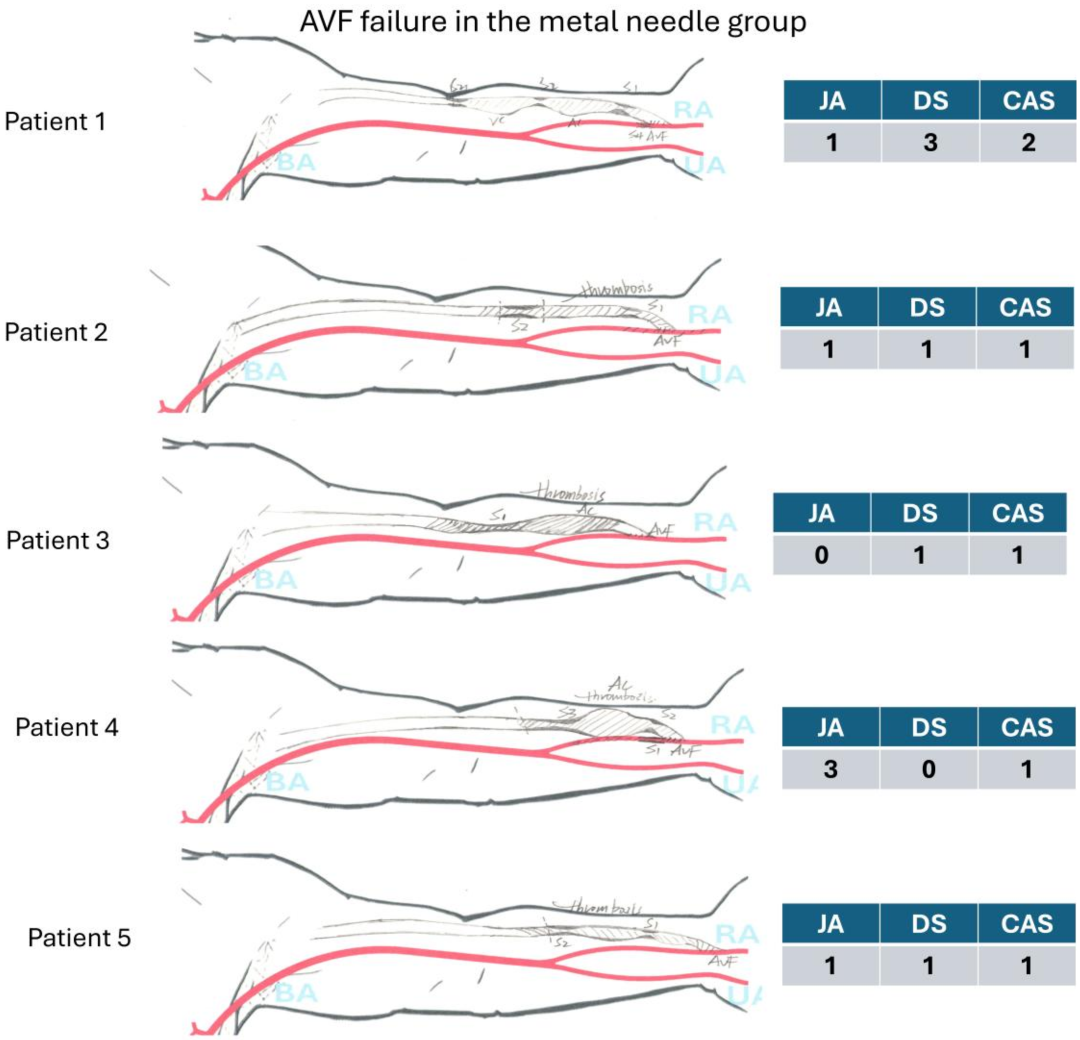

Figure 6. Anatomical locations of stenosis in five patients from the metal needle group. Schematic shows stenotic sites (S, numbered sequentially) in the juxta-anastomotic (JA) region, distal outflow (DS), and cannulation area stenosis (CAS).

AVF failure in the metal needle group

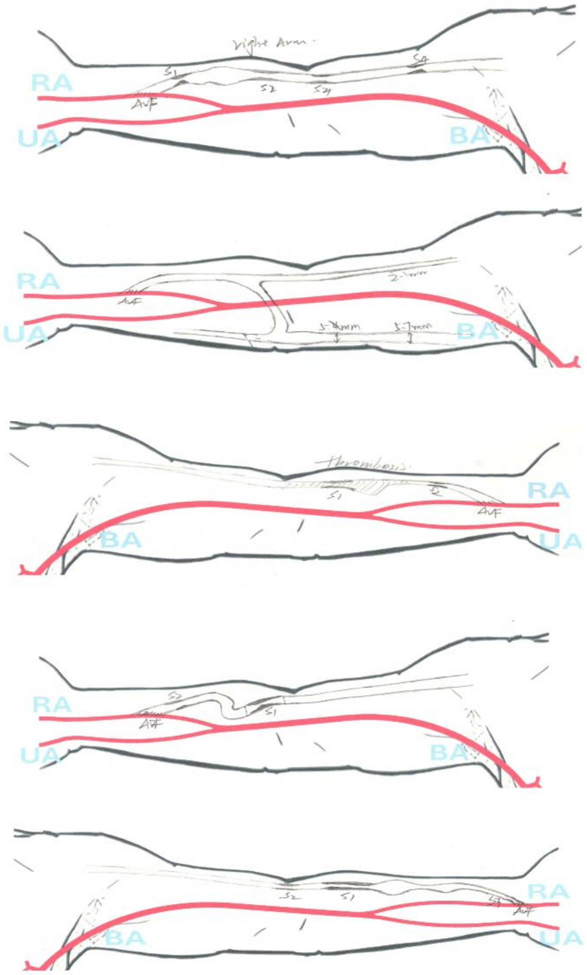

Patient 6

JA	DS	CAS
1	3	2

Patient 7

JA	DS	CAS
0	1	0

Patient 8

JA	DS	CAS
1	1	1

Patient 9

JA	DS	CAS
1	1	1

Patient 10

JA	DS	CAS
1	2	1

Figure 7. Anatomical locations of stenosis in five patients from the metal needle group. Schematic shows stenotic sites (S, numbered sequentially) in the juxta-anastomotic (JA) region, distal outflow (DS), and cannulation area stenosis (CAS).

Materials and Methods

1. Patient enrollment criteria

Inclusion criteria were as follows: (1) age > 18 years; (2) cephalic vein diameter at the puncture site > 4.5 mm and blood flow > 450 mL/min at first cannulation; (3) first-time AVF creation; (4) radio-cephalic or brachio-cephalic AVF configuration; (5) complete documentation of the first cannulation; (6) follow-up duration > 3 months; (7) availability of sufficient peri-cannulation blood samples (Timepoints 1 and 2); (8) complete baseline and endpoint ultrasonographic data (Timepoints 3 and 4); and (9) successful placement of two dialysis needles during the first cannulation.

In our hemodialysis center, physicians were more likely to recommend plastic cannulas during the initial cannulation period for patients with one or more of the following characteristics: (1) female and aged > 70 years; (2) poorly controlled diabetes; (3) cephalic vein diameter at the puncture site < 4.9 mm; (4) history of ipsilateral deep vein catheterization; (5) high pain sensitivity; (6) marked anxiety toward cannulation; or (7) strong personal preference.

2. The procedure to follow up

The AVFs were created and monitored by four HD physicians, all certified in surgery, across three HD centers. Follow-up interventions were provided as necessary. The follow-up period spanned from March 1, 2020, to December 31, 2024. After AVF creation, patients were followed at 1, 3, 5, and 7 days, 2, 3, and 4 weeks, and every 1–2 months thereafter, depending on vascular status and fistula functionality. If the AVF

became occluded, patients could visit the physicians for additional care. Vein diameter and access blood flow rate were measured using ultrasonography.

7. Surgical procedure

Radial-cephalic and brachial-cephalic AVFs were created following established protocols, using brachial plexus block or local anesthesia, along with local intra-arterial and intravenous heparin administration at a 1:100 ratio. For the brachial-cephalic AVF, an end-to-side anastomosis was performed between the cephalic vein and the radial artery at the wrist or forearm, with an arteriotomy size of 1 cm. For the brachial-cephalic AVF, the anastomosis was made between the cephalic vein and the brachial artery at the elbow, with an arteriotomy size of 5 mm. All surgeries were conducted by the same surgical team, and all anastomoses were performed using a 7-0 polypropylene.

8. Sample size calculation

This study employed a retrospective design, with the plastic cannula group serving as the experimental arm and the metal needle group as the control. The primary endpoint was AVF failure. Based on prior data, the AVF failure rate (p_1) in the plastic cannula group was 0.039, compared to 0.351 in the metal needle group (p_2).[1] With a power of 90% ($1 - \beta = 0.9$) and a 1:1 allocation ratio, the required sample size per group was calculated using the method described by L. Douglas Case et al. [2] and implemented *via* R software. Accounting for an anticipated 10% rate of loss to follow-

up, a minimum of 33 patients per group was required.

9.The choice of First cannulation time (FCT)

FCT was defined as the interval between arteriovenostomy and the first successful cannulation. Successful cannulation was determined by the ability to perform cannulation with two needles for two consecutive weeks and to administer prescribed dialysis within the designated timeframe. Cannulation was performed when the access blood flow rate exceeded 450 mL/min, the vein diameter was greater than 4.5 mm, and the fistula was clearly visible, positioned directly beneath the skin. A senior nurse performed the cannulation using either 17-G plastic cannulas (external diameter = 1.5 mm; internal diameter = 1.067 mm; lengths = 30 mm and 38 mm) or 17-G metal needles (external diameter = 1.47 mm; internal diameter = 1.067 mm; lengths = 20 mm and 25 mm). The rope-ladder technique was employed for all AVF cannulations. Blood flow during the first HD session was maintained at 140–200 mL/min, with all patients undergoing dialysis three times per week.

10. Assessment with vascular Doppler ultrasonography

Intraoperative measurements were obtained immediately following arteriovenous anastomosis. Vessel diameters were determined as the vertical distance between the external walls, utilizing electronic measurement software from the vascular Doppler ultrasonography system. Simultaneously, hemodynamic parameters, including peak systolic velocity (PSV, cm/s) and mean velocity (Vm, cm/s), were recorded. The

cross-sectional area of the vessel was calculated assuming a circular shape, and blood flow (BF) was computed by multiplying Vm with the cross-sectional area using the Doppler ultrasound machine's software.

11. Microarray

Total RNA was extracted and purified using the RNeasy Micro Kit (Cat. #74004, QIAGEN, GmBH, Germany). The RNA integrity number (RIN) ≥ 7 and 28S/18S ≥ 0.7 were checked to inspect RNA integration using Agilent Bioanalyzer 2100 (Agilent Technologies). Total RNA was amplified and labeled with the Low Input Quick Amp Labeling Kit, One-Color (Cat. #5190-2305, Agilent Technologies). Labeled cRNA was purified with the RNeasy Mini Kit (Cat. #74106, QIAGEN, GmBH, Germany). Each slide was hybridized with 600 ng of Cy3-labeled cRNA using the Gene Expression Hybridization Kit (Cat. #5188-5242, Agilent Technologies) in a hybridization oven at 65°C (Cat. #G2545A, Agilent Technologies). After 17 hours of hybridization, the slides were washed in staining dishes (Cat. #121, Thermo Shandon, Waltham, MA, the USA) with the Gene Expression Wash Buffer Kit (Cat. #5188-5327, Agilent Technologies). The slides were scanned using the Agilent Microarray Scanner (Cat. #G2565CA, Agilent Technologies) with default settings: dye channel: green, scan resolution = 3 μm, photomultiplier tube (PMT) 100%, and 20 bit. Data were extracted with the Feature Extraction software 10.7 (Agilent Technologies, Santa Clara, CA, the USA).

12. Blood sampling and assays

Blood samples were drawn from the HD vascular access prior to dialysis and heparin administration, with a 9:1 volume ratio of blood to sodium citrate (0.129 mol/L). Complement antigen levels were quantified using enzyme-linked immunosorbent assay (ELISA) kits (Biomatik, Canada) for C1q (EKN44480), C1r (EKU09849), C1s (EKC41401), C3a (EKF57555), C5a (EKF57019), C8a (EKC33262), C9 (EKF59021), CFB (EKL56235), CFD (EKF58966), MASP2 (EKC41327), MBL (EKN46900), and C5b-9 (EKE62531).

13. Immunofluorescence

Deparaffinized human AVF outflow access sections were incubated with 4% fetal bovine serum in phosphate-buffered saline (PBS) for 30 minutes, followed by co-incubation with mouse anti-human CFB (1:50, BF8079) and rabbit anti-human C5b-9 (1:50, Ab55811) antibodies overnight at 4°C. Secondary antibodies were applied based on the primary antibody origin: Alexa 647-conjugated goat anti-mouse IgG H&L (1:200, ab150115) and Alexa 647-conjugated donkey anti-rabbit IgG H&L (1:200, ab150075). Samples were mounted using Prolong® Gold Antifade with DAPI (blue). Visualization was performed using a Leica TCS SP5 confocal scanning laser microscope (Leica Microsystems, IL, USA) and LAS AF Lite (version 2.6). Merged red and blue images were generated using Image Pro Plus (version 6.0).

14. Western blot

The isolated cells were homogenized in freshly prepared tissue protein extraction reagent (Pierce Bioscience, Rockford, IL, USA). After centrifugation, the supernatants were stored at –80°C. Primary antibodies used included anti-eNOS (ab252439, 1:1000, Abcam) and anti-GAPDH (ab8245, 1:3000, Abcam).

15. qRT-PCR

For qRT-PCR, the SYBR Green PCR Master Mix (Toyobo, Osaka, Japan) was used with the Rotor-Gene 3000A system (Corbett, Sydney, Australia) following the manufacturers' protocols. Briefly, each 20 μL PCR reaction contained 2 μL cDNA, 0.4 μL of both forward and reverse primers, and 10 μL SYBR Green I. After an initial denaturation at 95°C for 1 minute, the reaction was cycled 45 times, with each cycle consisting of denaturation at 95°C for 15 seconds, followed by primer annealing and extension at 60°C for 31 seconds. Gene expression levels were normalized to *Gapdh* expression and presented as relative expression. The primer sequences included: NOS3, (F) TGA TGG CGA AGC GAG TGA AG, (R) ACT CAT CCA TAC ACA GGA CCC; ICAM1, (F) TTG GGC ATA GAG ACC CCG TT, (R) GCA CAT TGC TCA GTT CAT ACA CC; CFB, (F) GCA CTG GAG TAC GTG TGT CC, (R) CCC GTT CTC GAA GTC GTG TG; S100A8, (F) ATG CCG TCT ACA GGG ATG AC, (R) ACT GAG GAC ACT CGG TCT CTA; SMAD2, (F) CCG ACA CAC CGA GAT CCT AAC, (R) GAG GTG GCG TTT CTG GAA TAT AA; ARG1, (F) CCC TGG GGA ACA CTA CAT TTT G, (R) GCC AAT TCC TAG TCT GTC CAC TT; CXCR3, (F) TTT GAC CGC TAC CTG AAC ATA GT, (R) GGG AAG TTG TAT TGG CAG

TGG; TNF, (F) GAG GCC AAG CCC TGG TAT G, (R) CGG GCC GAT TGA TCT CAG C.

16. *In vitro* experiments

(1) EdU assay for cell proliferation

The proliferation of EA.hy 926 cells (ATCC® CRL-2922™, ATCC, USA) was evaluated using Molecular probes® Click-iT® EdU image kit (Invitrogen TM, Carlsbad, CA, USA). First, the cells were plated on coverslips overnight and 2×EdU working solution was used to dilute the media containing cells. Then the cells were incubated for 4 h followed by immediate fixation and permeabilization. The Click-iT reaction cocktail (0.5 mL) was added to each well with a coverslip and incubated for 30 min in the dark. DNA was stained with Hoechst 33342 and the samples were imaged via Olympus BX-51 fluorescence microscopy. Each well was washed twice with 1 mL PBS between the two steps.

(2) 5(6)-Carboxyfluorescein N-hydroxysuccinimidyl ester (CFSE) assay

For the cell proliferation assay, CFSE labeling kit (#ab113853, Abcam) was used according to the manufacturer's recommendations. Briefly, we firstly incubated EA.hy 926 cells with 5 μM CFSE in PBS at 37°C for 15 min. Next, we terminated the reaction with cold DMEM supplemented with 5% FBS and stood the tube at 4°C for 5 min. Then we pelleted the cells at 1000 rpm at 4°C and washed the cell deposits with DMEM again. Finally, we planted the cells into the proliferative system.

(3) Detection and measurement of NO production

An equal number of cells (5×10^5) were cultured in 60-mm dishes for 24 h. Culture medium was collected for measurements of NO levels. A NO analyzer (NOA 280i, Sievers Instruments, USA) was used for determination of endogenous NO levels. Aliquots of culture medium were injected into a nitrogen-purged chamber containing a reducing agent, which converts the NO oxidizing product nitrate back to NO. Steady-state micromolar concentrations of NO were calculated from the peak areas of absolute NO detected and compared with a sodium nitrite standard curve as reference. Levels of NO detected in the reaction medium were corrected for background noise by subtracting the amount of NO present in F-12 supplemented with 10% FBS.

References

1. Zhang D, Kong D, Ma L, Yang Y. Using a plastic cannula prevents arteriovenous fistula failure in early cannulation. Ren Fail.2025;47:2458192.
2. Case LD, Ambrosius WT. Power and sample size. Methods Mol Biol.2007;404:377-408.
